# The effects of nonpharmacological sleep hygiene on sleep quality in nonelderly individuals: A systematic review and network meta-analysis of randomized controlled trials

**DOI:** 10.1371/journal.pone.0301616

**Published:** 2024-06-05

**Authors:** Kenta Hirohama, Takeshi Imura, Tomonari Hori, Naoki Deguchi, Tsubasa Mitsutake, Ryo Tanaka

**Affiliations:** 1 Graduate School of Humanities and Social Sciences, Hiroshima University, Hiroshima, Japan; 2 Department of Rehabilitation, Sakamidorii Hospital, Hiroshima, Japan; 3 Department of Rehabilitation, Faculty of Health Sciences, Hiroshima Cosmopolitan University, Hiroshima, Japan; 4 Department of Rehabilitation, Fukuyama Rehabilitation Hospital, Fukuyama, Japan; 5 Research Team for Promoting Independence and Mental Health, Tokyo Metropolitan Institute for Geriatrics and Gerontology, Itabashi, Tokyo, Japan; 6 Clinical Research Center, Saga University Hospital, Saga, Japan; UNAM Facultad de Estudios Superiores Zaragoza: Universidad Nacional Autonoma de Mexico Facultad de Estudios Superiores Zaragoza, MEXICO

## Abstract

The prevalence of locomotive syndrome naturally increases with age, but approximately half of nonelderly individuals also meet the criteria for locomotive syndrome, suggesting that even younger people need to pay attention to their own health status. Sleep is important for physical, cognitive, and psychological health. Some individuals with poor sleep quality may be at risk of developing negative health status. Although the effects of sleep hygiene strategies for elderly individuals have been well investigated, optimal nonpharmacological sleep hygiene strategies for improving sleep quality in nonelderly individuals has not been identified. We conducted a systematic review and network meta-analysis (NMA) of randomized controlled trials aimed to elucidate the effects of various nonpharmacological interventions on sleep quality in nonelderly individuals and to identify the optimal intervention. Cochrane Central Register of Controlled Trials, Medline, Cumulative Index to Nursing and Allied Health Literature, Physiotherapy Evidence Database, and Scopus were comprehensively searched. We identified 27 studies focusing on the effects of various nonpharmacological sleep hygiene strategies in nonelderly individuals, and 24 studies were applied into NMA. The present results showed that resistance training was the most effective intervention for improving sleep quality in nonelderly individuals. In addition, this study revealed the effects of nonpharmacological interventions, such as physical activity, nutritional intervention, as well as exercise interventions. This is the first report that utilized NMA to compare the effects of various nonpharmacological interventions on sleep quality in nonelderly individuals.

## Introduction

The prevention of musculoskeletal dysfunction and improvement of functional recovery after musculoskeletal diseases are necessary to maintain the long-term quality of life of people of various generations. Locomotive syndrome, first identified by the Japanese Orthopaedic Association in 2007, includes various impairments as compared to musculoskeletal gait dysfunction [1]. The prevalence of locomotive syndrome naturally increases with age, but approximately 40% to 50% of nonelderly individuals in their 40s and 50s also meet the criteria for locomotive syndrome [2]. More surprisingly, 21.7% of men and 25% of women aged < 40 years meet the criteria for locomotive syndrome [2], suggesting that even younger people need to pay attention to their own health status.

Frailty is considered an age-related biological syndrome characterized by a decreased biological reserve capacity due to changes in several physiological systems and decreased resistance to stressors, exposing individuals to the risk of negative outcomes (disability, falls, hospitalization, and death) due to minor stressors [3–7]. Frailty is also prevalent in nonelderly patients, especially those undergoing surgery, and is associated with an increased risk of postoperative hospital mortality [8, 9]. Locomotive syndrome and frailty are not unavoidable events that always occur with aging. In fact, adults who continue to practice a healthy lifestyle, avoid inactivity, participate in physical exercise (walking, strength training, physical activity, etc), use care prevention services, and engage with family and friends tend to maintain healthy, independent lives and reduce health-related costs [10], possibly indicating that these negative events, such as locomotive syndrome or frailty, can be prevented, postponed, or even ameliorated by optimal interventions at appropriate timing [11].

Sleep problems and frailty have been suggested to have a relationship in adults [12]. Frailty reportedly can lead to disrupted sleep cycles, and a bilateral relationship between frailty and sleep problems has also been proposed [13]. Poor sleep quality is a common problem in adults with an estimated prevalence ranging from 12% to 40% [14, 15]. Poor sleep quality is associated with cognitive impairment [16], decreased quality of life [17, 18], and economic burden [19]. Roncoroni et al. have investigated the effects of sleep deficiencies in nonelderly individuals and reported that worse sleep quality was associated with a higher likelihood of developing negative health status, including being overweight, often feeling depressed, or often feeling anxious [20]. To achieve healthy aging, sleep quality improvement is considered an important health promotion strategy. Additionally, sleep problems, such as poor sleep quality, are among the most common comorbidities associated with various musculoskeletal pain [21–24]. The prevalence of insomnia is twice as great in patients with osteoarthritis (OA) (25%) as compared to those without OA (11%). More than two-thirds of OA patients have sleep disturbances [25]. Poor sleep quality is associated with musculoskeletal pain and may be a risk factor for locomotive syndrome and physical frailty.

Sleep is important for physical, cognitive, and psychological health, but many people do not have a good sleep [26–28]. In fact, 50 and 70 million American adults have sleep problems, and one-thirds of adults do not get enough sleep [29, 30]. More than 9 million U.S. adults aged ≥ 30 years depend on sleep medication to fall asleep each night [31, 32]. Middle-aged and older adults are more likely to take medication for sleep support due to age-related decline in sleep quality and duration [33]. A number of nonpharmacological alternatives to improve sleep quality, such as cognitive behavioral therapy, mindfulness meditation, and physical activity, exist [14, 34, 35]. Some individuals with poor sleep quality may be at risk of becoming frail earlier in life or in the future [36–38]. Thus, effective early sleep hygiene strategies may help to reduce future risk in nonelderly individuals.

The European Guideline for the Diagnosis and Treatment of Insomnia states that exercise is effective in the management of insomnia [39] and suggests that exercise may also be effective in improving sleep quality. A recent network meta-analysis (NMA) has reported that a combination of aerobic exercise and resistance training, as well as exercise under face-to-face supervision, is effective for improving the sleep quality in older adults [40]. However, the optimal sleep hygiene strategies for improving sleep quality in nonelderly individuals has not been identified. A relatively new analysis method, NMA, allowed a direct and an indirect comparison of multiple interventions to determine the relative effectiveness of various interventions. Here, we aimed to elucidate the effects of various interventions on sleep quality in nonelderly individuals and to identify the optimal intervention by conducting a systematic review and NMA of randomized controlled trials (RCTs).

## Materials and methods

This systematic review and NMA are reported according to the Preferred Reporting Items for Systematic Reviews and Meta-Analyses statement [41], and was registered in UMIN-CTR (ID: UMIN000050666).

### Literature search

Following electronic databases were searched from the earliest records to February 21, 2023: Cochrane Central Register of Controlled Trials (CENTRAL), Medline (PubMed), Cumulative Index to Nursing and Allied Health Literature, Physiotherapy Evidence Database (PEDro), and Scopus. Further details about the search strategy are provided in the S1 File.

### Selection criteria

Inclusion criteria for studies were as follows: 1) healthy individuals who had not been diagnosed with any diseases by a physician; 2) the mean reported age of the participants was ≤64 years; 3) use of nonpharmacological interventions aimed to improve sleep quality and quantity (nutritional interventions, lifestyle modification, exercise, physical activity, etc); 4) sleep quality and quantity were quantitatively assessed (including subjective and objective assessments); 5) the study design was RCT; and 6) publication in a peer-reviewed journal. The exclusion criteria included the following: 1) pregnant or postpartum women as participants; 2) the participants had sleep disorders; 3) the participants used medications; 4) nonpharmacological interventions other than sleep hygiene were performed; and 5) no descriptive statistics to perform a meta-analysis.

### Study selection

Two reviewers independently conducted the search, screened the article titles, and reviewed the abstracts to assess eligibility (KH and TH). Articles that appeared to meet the eligibility criteria were included for consideration in the full-text review completed by two reviewers. The articles included in the systematic review were determined by consensus (KH and TH). Any disagreements during the article screening and article selection were mediated through a discussion with a third reviewer (TI).

### Data extraction

Using a standardized data extraction form, one reviewer (KH) initially extracted information on the study participants, interventions, and sleep outcomes. Then, the second reviewer (TH) scrutinized and validated the extracted data. The extracted data included the means (final value and change score), standard deviations, sample size, and 95% confidence intervals (95% CIs). When sufficient information was unavailable, data were estimated using the recommended methods in the Cochrane Handbook for Systematic Reviews of Interventions [42].

### Risk of bias evaluation

Two independent reviewers (KH and TH) rated all included studies for the risk of bias using the Cochrane Risk of Bias tool, and disagreements were resolved by a third reviewer (TI). This tool comprises five domains related to the trial’s internal and statistical validity and is rated on a three-point scale. The scale is a reliable and valid tool for assessing the risk of bias in individual trials.

### Network meta-analysis

The outcome data were extracted, and a NMA was conducted using the outcome values assessed immediately after the end of the intervention. The outcome data used in the NMA was sleep quality. Effect sizes for sleep quality comparisons were calculated with a random-effects model using Review Manager version 5.4 for standardized mean differences (SMD) and standard errors. We used a frequentist random-effects NMA model with restricted maximum likelihood estimation methods.

This frequentist framework was employed instead of a Bayesian framework, because frequentist NMA assumes a simple model and uninformative prior distributions for all intervention effect parameters, and the results of Bayesian and frequentist analyses are similar. The NMA was conducted using the netmeta package of the statistical software R (version 3.3.3). Heterogeneity was assessed using the Cochran Q test (significance level is P < 0.01) and I^2^ statistic (I^2^ > 50%).

Total heterogeneity in the network was evaluated by decomposing it into the following two components: heterogeneity within designs and discrepancy between designs. If significant heterogeneity was found, a Q value was calculated to indicate total inconsistency based on the full design-by-treatment interaction random-effects model [42]. Based on the indicated Q values, we decided whether to use a random-effects or a fixed-effects model (the model with the smaller Q value was adopted). If subnetwork formation was observed during modeling, the network structure was checked, and the analytical dataset was reduced to ensure that only the interventions connected to the network were included.

Forest plots were created to show the effects of various interventions as compared to the control. Additionally, net-heat plots were created to assess the contribution of the network model to the design’s inconsistency and inconsistency. Moreover, the P-score was used to assess relative effectiveness, as it measures the certainty that one intervention is better than the other, averaging over all competing interventions; the P-score has been shown to be equivalent to the SUCRA score [43].

### Grading of evidence

The evidence was evaluated using the Grading of Recommendations Assessment, Development and Evaluation for NMA (GRADE-NMA) [44]. Strength of evidence was assessed based on four domains: risk of bias, inconsistency, imprecision, and other confounding factors (including publication bias). Publication bias was evaluated by a comparison of adjusted funnel plots. Evidence quality was downgraded one level if a domain was rated “severe” and two levels if a domain was rated “very severe.” The decision to use direct, indirect, or network estimates was made according to the evaluation process reported by Izcovich et al. [44]. Based on this approach, the evidence was graded as high, medium, low, or very low.

## Results

**Fig 1:**
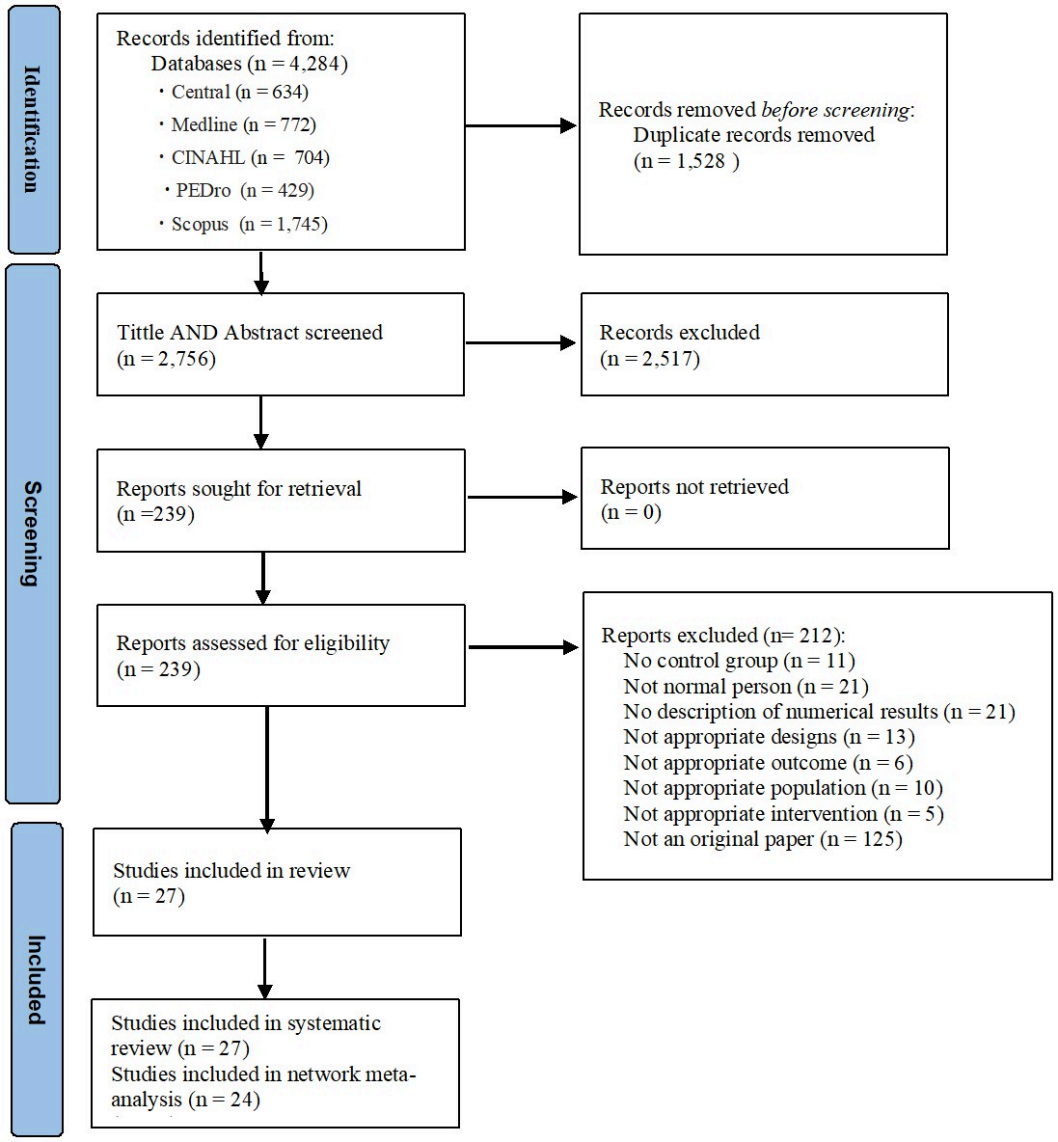
Flow diagram of the systematic review. Central, Cochrane Central Register of Controlled Trials; CINAHL, Cumulative Index to Nursing and Allied Health Literature, PEDro; Physiotherapy Evidence Database.

The literature search through 5 databases identified 4,284 articles (Fig 1). After removing duplicates and verifying the titles and abstracts, 239 full-text articles were retrieved and evaluated for eligibility. Altogether, 212 articles were excluded due to the inappropriate study design, intervention, population, or outcome, among results. As a result, 27 articles remained for the systematic review (Table 1 [45–71]). However, we excluded 3 articles (Ahmadinezhad et al., 2017 [66]. Akinci et al., 2022 [67]. Fenton et al., 2021. [53]) that consist of subnetwork during the NAM process. Finally, 24 articles were included in the NMA.

**Table 1:** Basic summary of included studies.

Author, year	Population	Intervention methods	Detailed intervention	Outcome of sleep quality	RoB
**Hudson JL, 2020. [** 45 **]**	Obesity	Nutritional intervention	Dietary Guidelines for Americans protein food group animal protein foods to provide 12.5 oz eq/day of protein.	Actigraph	Some concerns
**Nakashima A, 2020. [** 46 **]**	Poor sleep quality adult	Nutritional intervention	The Midori Mushi powder was consumed twice daily (after breakfast and dinner).	Validated questionnaire	Some concerns
**Oftedal S, 2019. [** 47 **]**	Poor sleep quality adult	Nutritional intervention and Pysical Activity	The application was used to set and self-monitor physical activity, diet quality, and sleep goals. Weekly summary reports were submitted.	PSQI and ESS	Some concerns
**Wilson D, 2022. [** 48 **]**	Obesity	Nutritional intervention and Physical Activity	Behavior Change Technology. Sleep hygiene, healthy eating, and physical activity goals were established.	PSQI	High
**Ha Y, 2022. [** 49 **]**	Healthy adult	Lifestyle Modification	Mobile wellness program and online exercise. Weeks 1–6: Goal 5000–9999 steps per day. Weeks 7–12: Goal Add 1000 steps per day every 2 weeks. Exercise intensity is 50–60% of target heart rate. Online exercise twice a week for 1 hour each.	PSQI	Some concerns
**Murawski B, 2019. [** 50 **]**	Poor sleep quality adult	Lifestyle Modification	Intervention by app and non-app app: educational resources, self-monitoring, goal setting, feedback. non-app: delivered via participant handbook, text message, email.	PSQI	Some concerns
**Murawski B, 2020. [** 51 **]**	Poor sleep quality adult	Lifestyle Modification	Encourage gradual engagement in the recommended weekly amount of physical activity for adults.	PSQI	High
**Martin CK, 2016. [** 52 **]**	Healthy adult	Lifestyle Modification	2 years of 25% calorie restriction.	PSQI	Some concerns
**Fenton S, 2021. [** 53 **]**	Obesity	Lifestyle Modification	Move, Eat & Sleep, a weight loss intervention with multiple behavior change.	PSQI and accelerometer	Some concerns
**Leonel LDS, 2022. [** 54 **]**	Obesity	Aerobic and Resistance Training	Combined aerobic and resistance training NG: remained at a moderate intensity throughout all study. PG: participated in a linear periodization training, divided into three mesocycles of five weeks each	PSQI	Some concerns
**Quist JS, 2019. [** 55 **]**	Obesity	Aerobic exercise	Bicycle: Self-selected intensity Bike to and from work or school. MOD: Exercise intensity 50% VO2peak-reserve. VIG: Exercise intensity 70% VO2peak-reserve.	PSQI and ESS	High
**Tseng TH, 2020. [** 56 **]**	Poor sleep quality adult	Aerobic exercise	Exercise Training Program Three times a week for 12 weeks 40 minutes of supervised aerobic exercise and 10 minutes of stretching class.	Actigraph and PSQI	Some concerns
**Niu SF, 2021. [** 57 **]**	Poor sleep quality adult	Aerobic exercise	Continuous walking on a treadmill 60 minutes, 3 times a week for 8 weeks. Exercise intensity is 60% to 80% of maximal heart rate.	Actigraph	Some concerns
**Elavsky S, 2007. [** 58 **]**	Insufficient exercise adults	Walking Yoga	Walking Program: One hour of moderate-intensity exercise, three times per week. Exercise duration started at 15 minutes and gradually increased to 60 minutes. Exercise intensity started at 50% of HRR and increased from 60% to 75%. Yoga Program: Low-intensity 90-minute meetings were held twice a week.	PSQI	Some concerns
**Barrett B, 2020. [** 59 **]**	Healthy adult	Meditation Walking	Mindfulness Meditation Training: The program took place once a week for two and a half hours for eight weeks. Practiced daily for 20 to 45 minutes. A five-hour weekend retreat was held during the sixth week. Exercise Reining.: Fast walking and jogging on a treadmill. Customized instruction was provided for those with physical limitations or who did not have access to specific equipment. Borg’s Perceived Exertion Rating, 12–16 points.	PSQI	Some concerns
**Atlantis E, 2006. [60]**	Healthy adult	Aerobic and Resistance training and Education	Various aerobic exercises of moderate to high intensity for 20 minutes at least 3 days per week. Moderate to high intensity full body weight training for 30 minutes at least 3 days per week. Health education seminars as a behavior change strategy.	PSQI	Some concerns
**Papp ME, 2019. [** 61 **]**	Healthy adult	Yoga	A standardized 1-hour HIY program was performed once a week for 6 weeks with an instructor.	PSQI and ISI	High
**Wang F, 2020. [** 62 **]**	Healthy adult	Walking	Participants in the IG were asked to perform DAWE The amount of intervention was targeted at 10,000 steps per day.	PSQI	High
**McDonough DJ, 2022. [** 63 **]**	Healthy adult	Aerobic and Resistance Training	Aerobic exercise and muscle strengthening PA (physical activity) videos were received weekly.	Actigraph	Some concerns
**Li M, 2015. [** 64 **]**	Healthy adult	Baduanjin	One hour of eight danjin exercise was practiced daily, five days a week.	PSQI	Some concerns
**Santiago LCS, 2022. [** 65 **]**	Healthy adult	Resistance Training	Perform 3 sets of 10–12 exercises of 8 different exercises.	PSQI	Some concerns
**Ahmadinezhad M, 2017. [** 66 **]**	Postmenopausal	Pilates Acupressure	PG: 3 sessions of 1 hour per week for 6 consecutive weeks. AG: Received acupressure intervention 3 sessions per week for 6 weeks.	PSQI	Some concerns
**Akinci B, 2022. [** 67 **]**	Insufficient exercise adults	Exercise	Video-conference group: Exercise sessions lasted 40–45 minutes, 3 days per week for 6 weeks, based on real-time movement demonstrations by the therapist, verbal explanations, and feedback to the subjects.	PSQI	Some concerns
**Genin PM, 2017. [** 68 **]**	Healthy adult	Physical activity	Twice weekly training, each session to be a minimum of 45 minutes, alternating between muscle strengthening exercises and aerobic breathing exercises. A group activity may be added as a third session each week.	ISI ESS	High
**Rayward AT, 2020. [** 69 **]**	Poor sleep quality adult	Sleep Hygiene and Physical Activity	Sleep intervention: reducing bed and wake time variability, engaging in a number of sleep hygiene behaviors stress management Physical Activity: increasing their daily minutes of MVPA and step counts and weekly RT	PSQI	High
**Tadayon M, 2016. [** 70 **]**	Postmenopausal	Physical activity	For 12 weeks, they engaged in moderate-intensity physical activity together twice a week at 6:00 p.m. for 1.5 hours. Activities included roller skating, biking, baseball, and walking/running.	PSQI	Some concerns
**Hurdiel R, 2017. [** 71 **]**	Healthy adult	Physical Activity	The subjects performed moderate-intensity physical activity for 1.5 hours, twice a week, for 12 weeks. Physical activities included roller skating, biking, baseball, and walking/running under supervision.	Actigraph PSQI	Some concerns

SQI, Pittsburgh Sleep Quality Index; ESS, Somnolence Scale: Epworth; NG, Non-periodized group; PG, Periodized group; MOD, Moderate intensity; VIG, Vigorous intensity; HRR, Heart Rate Reserve; HIY, Hatha yoga exercises; ISI, Insomnia Severity Index; DAWE, Daily aerobic walking exercise; PG, Pilates group; AG, Acupressure group; MVPA, moderate to vigorous physical activity; RT, Resistance training;

### Study characteristics

Altogether, 2,649 participants randomized to the intervention (n = 1,614) and control (n = 1,035) groups were included in the NMA (S1 Table). The sample sizes ranged from 19 to 495 participants (S2–S5 Tables). The mean reported age range was 16.0 to 62.2 years (16.4 to 61.1 years, intervention group; 16.0 to 62.2 years, control group); seven studies included adults with poor sleep quality, four studies included obese individuals, one study included postmenopausal women, and one study included adults with insufficient exercise. Moreover, 74.1% of the participants were women (1,963/2,649). The other basic characteristics of the participants or studies included in the NMA are shown in S1 Table. The exercise interventions included aerobic exercise, resistance training, walking, yoga, meditation, and baduanjin. Sleep quality was assessed by using the actigraph or self-report questionnaires, such as the Pittsburgh Sleep Quality Index and Epworth Sleepiness Scale. The additional details regarding the characteristics of the included studies are presented in S2–S5 Tables.

### Network plots

**Fig 2:**
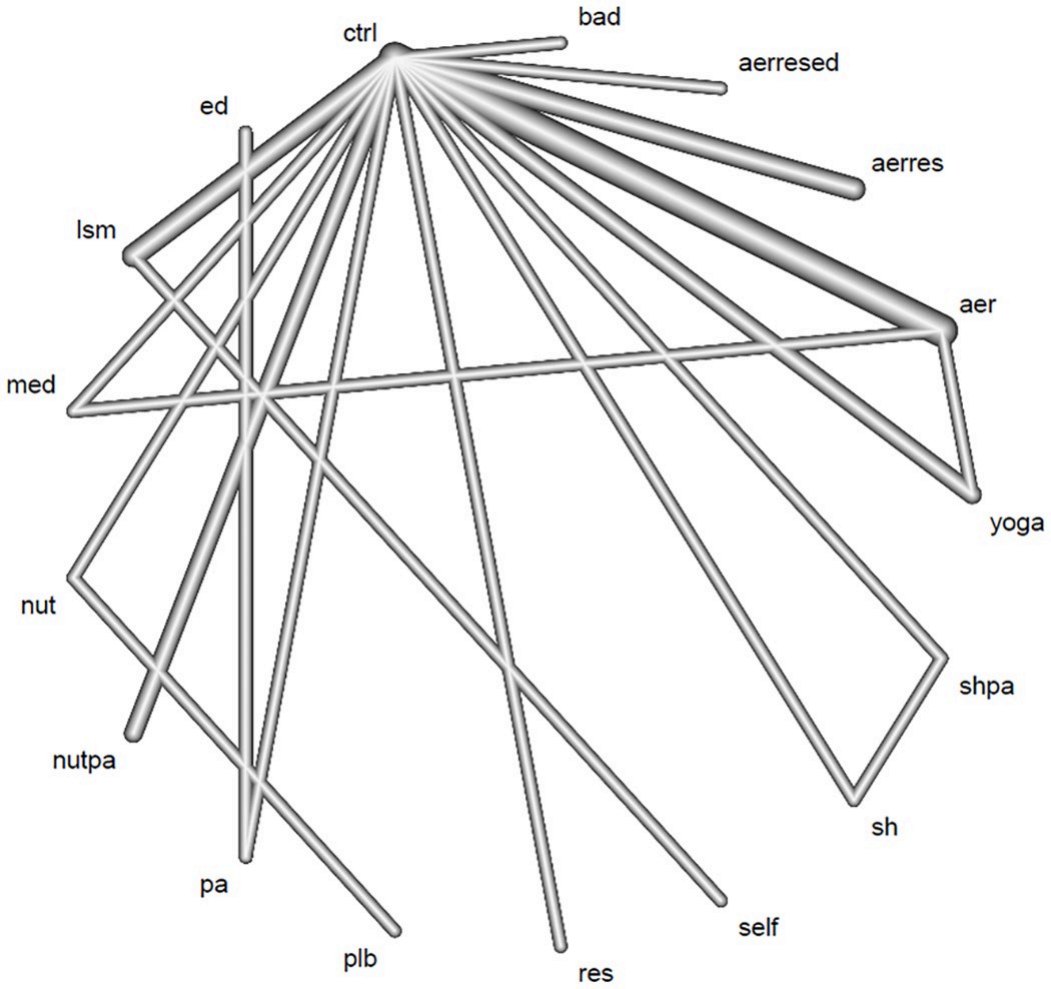
Network plot of comparison for sleep quality outcome. Aerobic exercise was the most frequently compared program among various interventions, and control was the most frequently compared one as control. aer, aerobic exercise; aerres, aerobic exercise and resistance training; aerresed, aerobic exercise, resistance training, and education; bad, baduanjin; ctrl, control: ed, education; lsm, lifestyle modification; med, meditation; nut, nutritional intervention; nutpa, nutritional intervention and pysical activity; pa, physical activity; plb, placebo; res, resistance training; self, self-monitor; sh, sleep hygiene; shpa, sleep hygiene and physical activity.

Fig 2 shows the network plots for sleep quality outcome. Each plot comprises 17 nodes, 24 direct comparisons, and 3 closed loops. Aerobic exercise was the most frequently compared program among study interventions, and control was the most frequently compared group.

### Effects of nonpharmacological various sleep hygiene interventions on sleep quality

**Fig 3:**
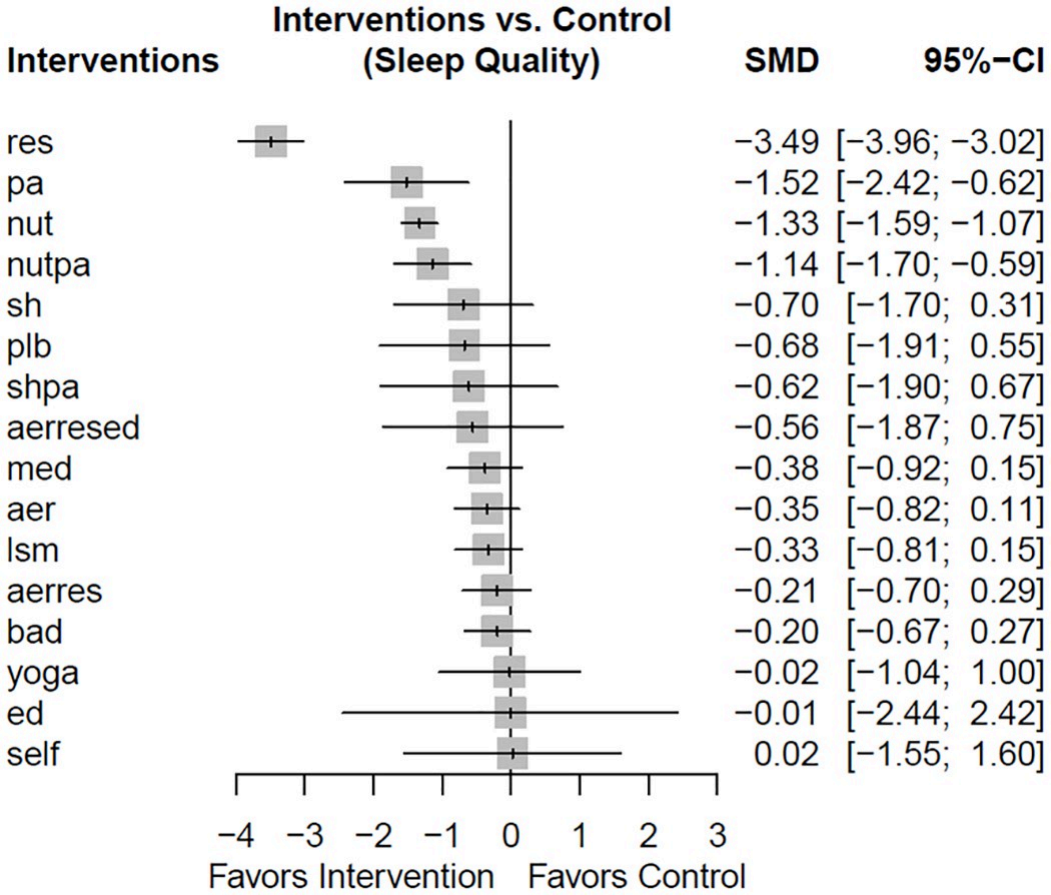
Forest plots displaying the standardized mean differences between control and various interventions for improving sleep quality. Resistance training, physical activity, nutritional intervention, the combination of nutritional intervention and physical activity were more effective in improving sleep quality than control. CI, confidence interval; aer, aerobic exercise; aerres, aerobic exercise and resistance training; aerresed, aerobic exercise, resistance training, and education; bad, baduanjin; ed, education; lsm, lifestyle modification; med, meditation; nut, nutritional intervention; nutpa, nutritional intervention and pysical activity; pa, physical activity; plb, placebo; res, resistance training; self, self-monitor; sh, sleep hygiene; shpa, sleep hygiene and physical activity; SMD, standardized mean differences.

Fig 3 summarizes the effects of various nonpharmacological interventions on sleep quality. The individuals with resistance training alone and physical activity alone had significantly improved sleep quality as compared with the control group (95% CI of SMD = −3.96 to −3.02, −2.42 to −0.62). Additionally, the individuals receiving nutritional intervention and a combination of nutritional intervention and physical activity had improved sleep quality as compared to the control group (95% CI of SMD = −1.59 to −1.07, −1.70 to −0.59). Interestingly, the 95% CI for resistance training (−3.96 to −3.02) did not overlap with the 95% CIs of other interventions, indicating that resistance training significantly improved sleep quality as compared with other interventions. According to the P-score results, resistance training had the highest score (0.99), followed by physical activity (0.85), nutritional intervention (0.83), and the combination of nutritional intervention and physical activity (0.76) (S6 Table).

### Inconsistency verification

To investigate the network consistency, a net-heat plot was created. No design-by-treatment inconsistencies or side-splitting inconsistencies were found (S1 Fig).

### Risk of bias evaluation in individual studies

Four studies had three items rated as having a high risk of bias (S7 Table). Three studies had two items rated as having a high risk of bias, and one study had one item rated as having a high risk of bias. Regarding overall judgment, seven studies were evaluated as having a high risk of bias, and the remaining 20 studies had some concerns.

### GRADE assessment

S8 Table presents the quality of evidence based on the GRADE system for the comparison of NMA. The quality of evidence ranged from very low to low.

## Discussion

The present systematic review elucidated the effects of various nonpharmacological interventions on sleep quality in nonelderly individuals. We identified 27 studies focusing on the effects of various sleep hygiene strategies in nonelderly individuals, and 24 studies were applied into NMA. Our data showed that resistance training was the most effective intervention for improving sleep quality in nonelderly individuals. It is very interesting to note that, unlike the results of NMA for older adults [40], our results included evidence on the effects of interventions, such as physical activity, nutritional intervention, as well as exercise interventions.

Regarding exercise intervention, resistance training alone was identified as the most effective intervention for improving sleep quality in nonelderly individuals. In a previous systematic review and NMA of elderly individuals, a combination of aerobic exercise and resistance training or exercise under face-to-face guidance were effective in improving sleep quality [40]. Kovacevic et al. have reported that resistance training may improve subjective sleep quality, with minimal effects observed on sleep quantity [72]. They also suggested that higher intensity and frequency of training may have a greater effect on sleep. In fact, in an RCT included in this NMA [65], a 55-minute resistance training intervention comprising three sets of 10–12 exercises of eight disciplines was performed three times a week, and they suggested that such a high training intensity and frequency had effects on sleep quality. The effect of resistance training on sleep quality is reportedly attenuated when combined with aerobic exercise [73]. In the elderly, resistance training alone might not result in a sufficient amount of load, and the combination of aerobic exercise might be effective. Whereas, in the nonelderly individuals, resistance training alone was sufficient to reach a sufficient workload, so the combination of aerobic exercise did not seem to have an additional effect.

The mechanisms by which exercise alters sleep and whether its effects are mediated in part by psychological, physiological, or neurophysiological changes are unknown. Resistance training may improve sleep, for example, by improving the symptoms of depression and anxiety, altering energy expenditure, increasing body temperature, and reducing musculoskeletal pain. Particularly, exercise is an effective intervention for depression, and sleep disturbances are among the core symptoms of depression. Thus, improvement in psychiatric symptoms may mediate some of the effects of exercise on sleep [74].

Physical activity in different types of exercise is thought to benefit sleep quality [75]. The RCT included in the NMA comprised an intervention of moderate-intensity physical activity for 1.5 hours twice a week at 6:00 p.m., in accordance with the World Health Organization recommendations, for 12 weeks, increasing the number of steps by 500 steps each week, reaching a maximum of 10,000 steps per day at the end of 12 weeks [71]. Physical activity interventions with positive results were similar to SR, which was reported to be primarily moderate or moderate-to-vigorous exercise [74].

The nutritional intervention or the combination of nutritional intervention and physical activity have also improved sleep quality. Many neurotransmitters are associated with the sleep—wake cycle, including serotonin, gamma-aminobutyric acid, orexin, melatonin, galanin, noradrenaline, and histamine [76]. Dietary precursors affect the rate of synthesis and function of a small number of neurotransmitters, including serotonin [77]. Serotonin synthesis can affect sleep and depends on the availability of its precursor, the amino acid L-tryptophan, in the brain. Combining tryptophan, a protein source, with carbohydrates improves sleep in patients with insomnia [78]. Additionally, ingestion of certain proteins that are high in tryptophan reportedly increases the availability of tryptophan and improve the sleep-related outcomes [79]. Regarding sleep quality improvement with the ingestion of *Euglena*, a wide variety of nutrients, including vitamins, minerals, amino acids, and unsaturated fatty acids, are supplied to the body, which not only improves the autonomic nervous system functioning but also normalizes the secretion of hormones and neurotransmitters, resulting in favorable effects on psychological status and sleep quality [46]. These findings might suggest the importance of nutritional interventions acting on various neurotransmitters in the brain and its role in altering sleep quality.

There are several limitations to the present study. First, the variations of individual study characteristics, including participant characteristics, sample size, or intervention protocol), might affect the study’s internal validity. Second, the number of studies examining the effects of some interventions, such as yoga, baduanzine, and meditation, were limited, which might affect the statistical power of the NMA. Third, the durations of each intervention included in this study widely varied. Therefore, it is impossible to discuss how long the effect of the intervention will emerge or persist on sleep quality. Fourth, there were concerns regarding the risk of bias for all articles included in the systematic review. As this could affect interpretation of the results, it should be considered as a limitation of the study. Finally, the quality of evidence was low and confidence in the effect estimates was limited. As true effects may differ substantially from effect estimates, they should be interpreted cautiously. Despite these certain limitations, to the best of our knowledge, this is the first report that utilized NMA to compare the effects of various nonpharmacological interventions on sleep quality in nonelderly individuals.

## Supporting information

S1 File Search strategies. (PDF)

S1 Table Basic characteristics of the participants or studies included in the network meta-analysis. (PDF)

S2 Table Detailed summary of nutritional intervention. (PDF)

S3 Table Detailed summary of lifestyle modification. (PDF)

S4 Table Detailed summary of exercise. (PDF)

S5 Table Detailed summary of physical activity. (PDF)

S6 Table P-score. (PDF)

S7 Table Risk of Bias. (PDF)

S8 Table GRADE assessment. (PDF)

S1 Fig A net-heat plot. (PDF)

S1 Checklist PRISMA NMA checklist of items to include when reporting a systematic review involving a network meta-analysis. (DOCX)
