# Supplementary material for: The effects of nonpharmacological sleep hygiene on sleep quality in nonelderly individuals: A systematic review and network meta-analysis of randomized controlled trials
Source: PLoS One. 2024 Jun 5;19(6):e0301616. doi: 10.1371/journal.pone.0301616 (PMC11152306; doi:10.1371/journal.pone.0301616)
Supplement: S1 File — (PDF) [file pone.0301616.s001.pdf]

Supplementary material

Search Strategies

Pubmed Search Strategy

#5 (#3) AND (#4)

#4 (#1) OR(#2)

#3 ("sleep"[MeSH Terms] OR “ Sleeping Habits “ [Title/Abstract] OR “ Sleep Habits “ [Title/Abstract] OR “ Habit, Sleep “ [Title/Abstract] OR “ Habits, Sleep “ [Title/Abstract] OR “Sleep Habit “ [Title/Abstract] OR “ Sleeping Habit “ [Title/Abstract] OR “ Habit, Sleeping “ [Title/Abstract] OR “ Habits, Sleeping “ [Title/Abstract]) OR ("sleep*"[Title/Abstract] AND "management*"[Title/Abstract]) OR ("sleep hygiene"[MeSH Terms] OR “ Hygiene, Sleep “ [Title/Abstract] OR “ Good Sleep Habits “ [Title/Abstract] OR “ Good Sleep Habit “ [Title/Abstract] OR “ Habit, Good Sleep “ [Title/Abstract] OR “ Habits, Good Sleep “ [Title/Abstract] OR “ Sleep Habit, Good “ [Title/Abstract] OR “ Sleep Habits, Good “ [Title/Abstract]) OR ("wearable electronic devices"[MeSH Terms] OR “ Device, Wearable Electronic “ [Title/Abstract] OR “Electronic Device, Wearable “ [Title/Abstract] OR “ Wearable Electronic Device “ [Title/Abstract] OR “ Wearable Technology “ [Title/Abstract] OR “ Technology, Wearable “ [Title/Abstract] OR “ Wearable Technologies “ [Title/Abstract] OR “ Wearable Devices “ [Title/Abstract] OR “ Device, Wearable “ [Title/Abstract] OR “ Wearable Device “ [Title/Abstract] OR “ Electronic Skin “ [Title/Abstract] OR “ Skin, Electronic “ [Title/Abstract]) OR ("polysomnography"[MeSH Terms] OR “ Polysomnographies “ [Title/Abstract] OR “ Monitoring, Sleep “ [Title/Abstract] OR “ Sleep Monitoring “ [Title/Abstract] OR “ Somnography “ [Title/Abstract] OR “ Somnographies “ [Title/Abstract]) OR ("Actigraph"[Title/Abstract])

#2 ("exercise"[MeSH Terms] OR “ Exercises “ [Title/Abstract] OR “ Physical Activity “ [Title/Abstract] OR “ Activities, Physical “ [Title/Abstract] OR “ Activity, Physical “ [Title/Abstract] OR “ Physical Activities “ [Title/Abstract] OR “ Exercise, Physical “ [Title/Abstract] OR “ Exercises, Physical “ [Title/Abstract] OR “ Physical Exercise “ [Title/Abstract] OR “ Physical Exercises “ [Title/Abstract] OR “ Acute Exercise “ [Title/Abstract] OR “ Acute Exercises “ [Title/Abstract] OR “ Exercise, Acute “ [Title/Abstract] OR “ Exercises, Acute “ [Title/Abstract] OR “ Exercise, Isometric “ [Title/Abstract] OR “ Exercises, Isometric “ [Title/Abstract] OR “ Isometric Exercises “ [Title/Abstract] OR “ Isometric Exercise “ [Title/Abstract] OR “ Exercise, Aerobic “ [Title/Abstract] OR “ Aerobic Exercise “ [Title/Abstract] OR “ Aerobic Exercises “ [Title/Abstract] OR “ Exercises, Aerobic “ [Title/Abstract] OR “ Exercise Training “ [Title/Abstract] OR “ Exercise Trainings “ [Title/Abstract] OR “ Training, Exercise “ [Title/Abstract] OR “ Trainings, Exercise “ [Title/Abstract]) OR ("walking"[MeSH Terms] OR “ Ambulation“ [Title/Abstract]) OR ("exercise therapy"[MeSH Terms] OR “ Remedial Exercise “ [Title/Abstract] OR “ Exercise, Remedial “ [Title/Abstract] OR “ Exercises, Remedial

“ [Title/Abstract] OR “ Remedial Exercises “ [Title/Abstract] OR “ Therapy, Exercise “ [Title/Abstract] OR “ Exercise Therapies “ [Title/Abstract] OR “ Therapies, Exercise “ [Title/Abstract] OR “ Rehabilitation Exercise “ [Title/Abstract] OR “ Exercise, Rehabilitation “ [Title/Abstract] OR “ Exercises, Rehabilitation “ [Title/Abstract] OR “ Rehabilitation Exercises “ [Title/Abstract]) OR ("muscle stretching exercises"[MeSH Terms] OR “ Exercise, Muscle Stretching “ [Title/Abstract] OR “ Muscle Stretching Exercise “ [Title/Abstract] OR “ Static Stretching “ [Title/Abstract] OR “Stretching, Static “ [Title/Abstract] OR “ Active Stretching “ [Title/Abstract] OR “ Stretching, Active “ [Title/Abstract] OR “ Static-Active Stretching “ [Title/Abstract] OR “ Static Active Stretching “ [Title/Abstract] OR “ Stretching, Static-Active “ [Title/Abstract] OR “ Isometric Stretching “ [Title/Abstract] OR “ Stretching, Isometric “ [Title/Abstract] OR “ Ballistic Stretching “ [Title/Abstract] OR “ Stretching, Ballistic “ [Title/Abstract] OR “ Dynamic Stretching “ [Title/Abstract] OR “ Stretching, Dynamic “ [Title/Abstract] OR “ Proprioceptive Neuromuscular Facilitation (PNF) Stretching “ [Title/Abstract] OR “ PNF Stretching “ [Title/Abstract] OR “ PNF Stretchings “ [Title/Abstract] OR “ Stretching, PNF “ [Title/Abstract] OR “ PNF Stretching Exercise “ [Title/Abstract] OR “ Exercise, PNF Stretching “ [Title/Abstract] OR “PNF Stretching Exercises “ [Title/Abstract] OR “ Stretching Exercise, PNF “ [Title/Abstract] OR “ Proprioceptive Neuromuscular Facilitation “ [Title/Abstract] OR “ Neuromuscular Facilitation, Proprioceptive “ [Title/Abstract] OR “Proprioceptive Neuromuscular Facilitations “ [Title/Abstract] OR “ Passive Stretching “ [Title/Abstract] OR “ Stretching, Passive “ [Title/Abstract] OR “ Relaxed Stretching “ [Title/Abstract] OR “ Stretching, Relaxed “ [Title/Abstract] OR “Static-Passive Stretching “ [Title/Abstract] OR “ Static Passive Stretching “ [Title/Abstract] OR “Stretching, Static-Passive “ [Title/Abstract])

#1 "nutrition therapy"[MeSH Terms] OR “ Therapy, Nutrition “ [Title/Abstract] OR “Medical Nutrition Therapy” [Title/Abstract] OR “Nutrition Therapy, Medical” [Title/Abstract] OR “Therapy, Medical Nutrition” [Title/Abstract]

Cochrane Search Strategy

#1	MeSH descriptor: [Nutrition Therapy] explode all trees
#2	MeSH descriptor: [Exercise] explode all trees
#3	MeSH descriptor: [Walking] explode all trees
#4	MeSH descriptor: [Exercise Therapy] explode all trees
#5	MeSH descriptor: [Muscle Stretching Exercises] explode all trees
#6	((#2) OR (#3) OR (#4) OR (#5))
#7	((#1) OR (#6))
#8	MeSH descriptor: [Sleep] explode all trees
#9	((Sleep*) AND (management*)):ti,ab,kw
#10	MeSH descriptor: [Sleep Hygiene] explode all trees
#11	MeSH descriptor: [Wearable Electronic Devices] explode all trees
#12	MeSH descriptor: [Polysomnography] explode all trees
#13	MeSH descriptor: [Actigraphy] explode all trees
#14	((#8) OR (#9) OR (#10) OR (#11) OR (#12) OR (#13))

CINAL Search Strategy

S16 ((S1 OR S2 OR S3 OR S4 OR S5) AND (S12 AND S14)) AND (S13 AND S15)

S15 (S1 OR S2 OR S3 OR S4 OR S5) AND (S12 AND S14)

S14 S1 OR S2 OR S3 OR S4 OR S5

S13 (MH randomized controlled trials OR MH double-blind studies OR MH single-blind studies OR MH random assignment OR MH pretest-posttest design OR MH cluster sample OR TI (randomised OR randomized) OR AB (random*) OR TI (trial) OR (MH (sample size) AND AB (assigned OR allocated OR control)) OR MH (placebos) OR PT (randomized controlled trial) OR AB (control W5 group) OR MH (crossover design) OR MH (comparative studies) OR AB (cluster W3 RCT)) NOT ((MH animals+ OR MH animal studies OR TI animal model*) NOT MH human)

S12 S6 OR S7 OR S8 OR S9 OR S10 OR S11

S11 (MH "Actigraphy")

S10 (MH "Polysomnography")

S9 TI ((wearable electronic devices) OR (Device Wearable Electronic) OR (Devices Wearable Electronic) OR (Electronic Device Wearable) OR (Electronic Devices Wearable) OR (Wearable Electronic Device) OR (Wearable Technology) OR (Technologies Wearable) OR (Technology Wearable) OR (Wearable Technologies) OR (Wearable Devices) OR (Device Wearable) OR (Devices Wearable) OR (Wearable Device) OR (Electronic Skin) OR (Skin Electronic)) OR AB ((wearable electronic devices) OR (Device Wearable Electronic) OR (Devices Wearable Electronic) OR (Electronic Device Wearable) OR (Electronic Devices Wearable) OR (Wearable Electronic Device) OR (Wearable Technology) OR (Technologies Wearable) OR (Technology Wearable) OR (Wearable Technologies) OR (Wearable Devices) OR (Device Wearable) OR (Devices Wearable) OR (Wearable Device) OR (Electronic Skin) OR (Skin Electronic))

S8 (MH "Sleep Hygiene")

S7 TI ((Sleep*) AND (management*)) OR AB ((Sleep*) AND (management*))

S6 (MH "Sleep")

S5 AB ((Muscle Stretching Exercises) OR (Exercise, Muscle Stretching) OR (Muscle Stretching Exercise) OR (Static Stretching) OR (Stretching Static) OR (Active Stretching) OR (Stretching Active) OR (Static-Active Stretching) OR (Static Active Stretching) OR (Stretching Static-Active) OR (Isometric Stretching) OR (Stretching Isometric) OR (Ballistic Stretching) OR (Stretching Ballistic) OR (Dynamic Stretching) OR (Stretching Dynamic) OR (Proprioceptive Neuromuscular Facilitation (PNF) Stretching) OR (PNF Stretching) OR (PNF Stretchings) OR (Stretching PNF) OR (PNF Stretching Exercise) OR (Exercise PNF Stretching) OR (PNF

Stretching Exercises) OR (Stretching Exercise PNF) OR (Proprioceptive Neuromuscular Facilitation) OR (Neuromuscular Facilitation Proprioceptive) OR (Proprioceptive Neuromuscular Facilitations) OR (Passive Stretching) OR (Stretching Passive) OR (Relaxed Stretching) OR (Stretching Relaxed) OR (Static-Passive Stretching) OR (Static Passive Stretching) OR (Stretching Static-Passive)) OR TI ((Muscle Stretching Exercises) OR (Exercise, Muscle Stretching) OR (Muscle Stretching Exercise) OR (Static Stretching) OR (Stretching Static) OR (Active Stretching) OR (Stretching Active) OR (Static-Active Stretching) OR (Static Active Stretching) OR (Stretching Static-Active) OR (Isometric Stretching) OR (Stretching Isometric) OR (Ballistic Stretching) OR (Stretching Ballistic) OR (Dynamic Stretching) OR (Stretching Dynamic) OR (Proprioceptive Neuromuscular Facilitation (PNF) Stretching) OR (PNF Stretching) OR (PNF Stretchings) OR (Stretching PNF) OR (PNF Stretching Exercise) OR (Exercise PNF Stretching) OR (PNF Stretching Exercises) OR (Stretching Exercise PNF) OR (Proprioceptive Neuromuscular Facilitation) OR (Neuromuscular Facilitation Proprioceptive) OR (Proprioceptive Neuromuscular Facilitations) OR (Passive Stretching) OR (Stretching Passive) OR (Relaxed Stretching) OR (Stretching Relaxed) OR (Static-Passive Stretching) OR (Static Passive Stretching) OR (Stretching Static-Passive))

S4 (MM "Exercise Therapy: Joint Mobility (Iowa NIC)") OR (MM "Exercise Therapy: Balance (Iowa NIC)") OR (MM "Exercise Therapy: Ambulation (Iowa NIC)") OR (MM "Therapeutic Exercise+") OR (MM "Exercise Therapy: Muscle Control (Iowa NIC)")

S3 (MH "Walking")

S2 (MH "Exercise")

S1 AB ((Therapy Nutrition) OR (Medical Nutrition Therapy) OR (Nutrition Therapy Medical) OR (Therapy Medical Nutrition) OR (Previous Indexing) OR (Nutrition Therapy)) OR TI ((Therapy Nutrition) OR (Medical Nutrition Therapy) OR (Nutrition Therapy Medical) OR (Therapy Medical Nutrition) OR (Previous Indexing) OR (Nutrition Therapy))

PEDro Search Strategy

Abstract & Title : "sleep quality"
Therapy : non
Problem : non
Body Part : non
Subdiscipline : non
Topic : non
Method : Clinical trial
Author/Association : non
Title Only: non
Source: non
Published Since : non
New records added since: non
Score of at least: non
Return: 20 records at a time
When Searching: Match all search terms (AND)

Scopus Search Strategy

(((TITLE-ABS((Therapy Nutrition) OR (Medical Nutrition Therapy) OR (Nutrition Therapy Medical) OR (Therapy Medical Nutrition) OR (Previous Indexing) OR (Nutrition Therapy) OR (Dietary Supplements) OR (Dietary Supplement) OR (Supplements Dietary) OR (Dietary Supplementations) OR (Supplementations Dietary) OR (Food Supplementations) OR (Food Supplements) OR (Food Supplement) OR (Supplement Food) OR (Supplements Food) OR (Nutraceuticals) OR (Nutraceutical) OR (Nutriceuticals) OR (Nutriceutical) OR (Neutraceuticals) OR (Neutraceutical) OR (Herbal Supplements) OR (Herbal Supplement) OR (Supplement Herbal) OR (Supplements Herbal) OR (Proteins) OR (Protein) OR (Gene Products Protein) OR (Protein Gene Products) OR (Gene Proteins) OR (Proteins Gene) OR (Calcium) OR (Blood Coagulation Factor IV) OR (Coagulation Factor IV) OR (Factor IV Coagulation) OR (Calcium-40) OR (Calcium 40Factor IV) OR (Dietar* AND Variet*) OR (Vitamins) OR (Vitamin))) OR (TITLE-ABS((Life Style) OR (Life Styles) OR (Lifestyle) OR (Lifestyles) OR (Life Style) OR (Induced Illness) OR (Lifestyle Factors) OR (Factor Lifestyle) OR (Lifestyle Factor) OR (Healthy Lifestyle) OR (Lifestyle Healthy) OR (Lifestyles Healthy) OR (Healthy Life Styles) OR (Healthy Lifestyles) OR (Healthy Life StyleLife Style Healthy) OR (Life Styles Healthy))) OR (TITLE-ABS((Education) OR (Workshops) OR (Workshop) OR (Training Programs) OR (Program Training) OR (Programs Training) OR (Training Program) OR (Educational Activities) OR (Activities Educational) OR (Activity Educational) OR (Educational Activity) OR (Literacy Programs) OR (Literacy Program) OR (Program Literacy) OR (Programs Literacy) OR (Health Education) OR (Education Health) OR (Community Health Education) OR (Education Community Health) OR (Health Education Community) OR (Patient Education as Topic) OR (Education Patient) OR (Patient Education) OR (Education of Patients) OR (Mindfulness) OR (Cognitive Behavioral Therapy) OR (Behavioral Therapies Cognitive) OR (Behavioral Therapy Cognitive) OR (Cognitive Behavioral Therapies) OR (Therapies Cognitive Behavioral) OR (Therapy Cognitive Behavioral) OR (Psychotherapy Cognitive) OR (Therapy Cognitive) OR (Cognitive Therapies) OR (Therapies Cognitive) OR (Cognitive Therapy) OR (Cognitive Behaviour Therapy) OR (Behaviour Therapies Cognitive) OR (Behaviour Therapy Cognitive) OR (Cognitive Behaviour Therapies) OR (Therapies Cognitive Behaviour) OR (Therapy Cognitive Behaviour) OR (Cognitive Psychotherapy) OR (Cognitive Psychotherapies) OR (Psychotherapies Cognitive) OR (Cognition Therapy) OR (Cognition Therapies) OR (Therapies Cognition) OR (Therapy Cognitive Behavior) OR (Behavior Therapies Cognitive) OR (Cognitive Behavior Therapies) OR (Therapies Cognitive Behavior) OR (Therapy Cognition Behavior) OR (Therapy Cognitive) OR (Cognitive Behavior Therapy))) OR (TITLE-ABS((Exercise) OR (Physical Activity) OR (Activities Physical) OR (Activity Physical) OR (Physical Activities) OR (Exercise Physical) OR (Exercises Physical) OR (Physical Exercise) OR (Physical Exercises) OR (Acute Exercise) OR (Acute Exercises) OR (Exercise Acute) OR (Exercises Acute) OR (Exercise Isometric) OR (Exercises Isometric) OR (Isometric Exercises) OR (Isometric Exercise) OR (Exercise Aerobic) OR (Aerobic Exercise) OR (Aerobic Exercises) OR (Exercises Aerobic) OR (Exercise Training) OR (Exercise Trainings) OR (Training Exercise) OR (Trainings Exercise) OR (Running) OR (Runnings) OR (walking) OR (Ambulation) OR (exercise therapy) OR (Remedial Exercise) OR (Exercise Remedial) OR (Exercises Remedial) OR (Remedial Exercises) OR (Therapy Exercise) OR (Exercise Therapies) OR (Therapies Exercise) OR (Rehabilitation Exercise) OR (Exercise Rehabilitation) OR (Exercises Rehabilitation) OR (Rehabilitation Exercises) OR (Muscle Stretching Exercises) OR (Exercise, Muscle Stretching) OR (Muscle

Stretching Exercise) OR (Static Stretching) OR (Stretching Static) OR (Active Stretching) OR (Stretching Active) OR (Static-Active Stretching) OR (Static Active Stretching) OR (Stretching Static-Active) OR (Isometric Stretching) OR (Stretching Isometric) OR (Ballistic Stretching) OR (Stretching Ballistic) OR (Dynamic Stretching) OR (Stretching Dynamic) OR (Proprioceptive Neuromuscular Facilitation Stretching) OR (PNF Stretching) OR (PNF Stretchings) OR (Stretching PNF) OR (PNF Stretching Exercise) OR (Exercise PNF Stretching) OR (PNF Stretching Exercises) OR (Stretching Exercise PNF) OR (Proprioceptive Neuromuscular Facilitation) OR (Neuromuscular Facilitation Proprioceptive) OR (Proprioceptive Neuromuscular Facilitations) OR (Passive Stretching) OR (Stretching Passive) OR (Relaxed Stretching) OR (Stretching Relaxed) OR (Static-Passive Stretching) OR (Static Passive Stretching) OR (Stretching Static-Passive) OR (Resistance Training) OR (Training Resistance) OR (Strength Training) OR (Training Strength) OR (Weight-Lifting Strengthening Program) OR (Strengthening Program Weight-Lifting) OR (Strengthening Programs Weight-Lifting) OR (Weight Lifting Strengthening Program) OR (Weight-Lifting Strengthening Programs) OR (Weight-Lifting Exercise Program) OR (Exercise Program Weight-Lifting) OR (Exercise Programs Weight-Lifting) OR (Weight Lifting Exercise Program) OR (Weight-Lifting Exercise Programs) OR (Weight-Bearing Strengthening Program) OR (Strengthening Program Weight-Bearing) OR (Strengthening Programs Weight-Bearing) OR (Weight Bearing Strengthening Program) OR (Weight-Bearing Strengthening Programs) OR (Weight-Bearing Exercise Program) OR (Exercise Program Weight-Bearing) OR (Exercise Programs Weight-Bearing) OR (Weight Bearing Exercise Program) OR (Weight-Bearing Exercise Programs)))) AND ((TITLE-ABS((sleep) OR (Sleeping Habits) OR (Sleep Habits) OR (Habit Sleep) OR (Habits Sleep) OR (Sleep Habit) OR (Sleeping Habit) OR (Habit Sleeping) OR (Habits Sleeping) OR (Sleep* AND management*) OR (Sleep Hygiene) OR (Hygiene Sleep) OR (Good Sleep Habits) OR (Good Sleep Habit) OR (Habit Good Sleep) OR (Habits Good Sleep) OR (Sleep Habit Good) OR (Sleep Habits Good) OR (wearable electronic devices) OR (Device Wearable Electronic) OR (Devices Wearable Electronic) OR (Electronic Device Wearable) OR (Electronic Devices Wearable) OR (Wearable Electronic Device) OR (Wearable Technology) OR (Technologies Wearable) OR (Technology Wearable) OR (Wearable Technologies) OR (Wearable Devices) OR (Device Wearable) OR (Devices Wearable) OR (Wearable Device) OR (Electronic Skin) OR (Skin Electronic) OR (Polysomnography) OR (Polysomnographies) OR (Monitoring Sleep) OR (Sleep Monitoring) OR (Somnography) OR (Somnographies) OR (Actigraph))))) AND (((INDEXTERMS ("clinical trials" OR "clinical trials as a topic" OR "randomized controlled trial" OR "Randomized Controlled Trials as Topic" OR "controlled clinical trial" OR "Controlled Clinical Trials" OR "random allocation" OR "Double-Blind Method" OR "Single-Blind Method" OR "Cross-Over Studies" OR "Placebos" OR "multicenter study" OR "double blind procedure" OR "single blind procedure" OR "crossover procedure" OR "clinical trial" OR "controlled study" OR "randomization" OR "placebo")) OR (TITLE-ABS-KEY (("clinical trials" OR "clinical trials as a topic" OR "randomized controlled trial" OR "Randomized Controlled Trials as Topic" OR "controlled clinical trial" OR "Controlled Clinical Trials as Topic" OR "random allocation" OR "randomly allocated" OR "allocated randomly" OR "Double-Blind Method" OR "Single-Blind Method" OR "Cross-Over Studies" OR "Placebos" OR "cross-over trial" OR "single blind" OR "double blind" OR "factorial design" OR "factorial trial"))) OR (TITLE-ABS (clinical trial* OR trial* OR rct* OR random* OR blind*)))) AND (LIMIT-TO (DOCTYPE,"ar")) AND (LIMIT-TO (EXACTKEYWORD,"Randomized Controlled Trial"))
