# Supplementary material for: The effects of nonpharmacological sleep hygiene on sleep quality in nonelderly individuals: A systematic review and network meta-analysis of randomized controlled trials
Source: PLoS One. 2024 Jun 5;19(6):e0301616. doi: 10.1371/journal.pone.0301616 (PMC11152306; doi:10.1371/journal.pone.0301616)
Supplement: S1 Table — (PDF) [file pone.0301616.s002.pdf]

Supplementary Table 1 Basic characteristics of the participants or studies included in the network meta-analysis

Characteristics	
Total sample size, n (participants)	2,649
Intervention group	1,614
Control groups	1,035
Age, mean ± SD	37.61 ± 12.07
Intervention group	37.36 ± 12.17
Control	37.89 ± 12.24
Gender, %	
Male	25.9
Female	74.1
Population, n (articles)	
Obesity	4
Healthy adult	11
Poor sleep quality adult	7
Postmenopausal	1
Insufficient exercise adults	1
Intervention methods, n (articles)	
Exercise	12
Physical activity	4
Lifestyle modification	4
Nutrition	4
Intervention period, mean (week)	17.16

SD, Standard deviation
