# Supplementary material for: The effects of nonpharmacological sleep hygiene on sleep quality in nonelderly individuals: A systematic review and network meta-analysis of randomized controlled trials
Source: PLoS One. 2024 Jun 5;19(6):e0301616. doi: 10.1371/journal.pone.0301616 (PMC11152306; doi:10.1371/journal.pone.0301616)
Supplement: S2 Table — (PDF) [file pone.0301616.s003.pdf]

Supplementary Table 2 Detailed summary of nutritional intervention

Author, year	Population	Participant inclusion criteria	N I/C	Female (%) I/C	Mean age
					I/C
**Joshua L Hudson, 2020**	Obesity	Age: 30-69 years<br>BMI: 25.0-39.0<br>Weight stable (±3 kg for the past 3 months)<br>Not on an exercise program	21/30	80/90	48 (11) /50 (8)
**Nakashima A, 2020**	Poor sleep quality adult	Age: 20-64 years<br>Decreased motivation based on vitality as measured by the MOS 36-Item Short-Form Health Survey (SF-36).<br>Decreased sleep quality based on PSQI total score.	Placebo: 18<br>Euglena500: 19<br>Euglena1000: 20<br>Euglena3000: 21	Placebo: 55.6<br>Euglena500: 52.6<br>Euglena1000: 55<br>Euglena3000: 55	Placebo: 47.3 (9.5)<br>Euglena500: 47.8 (11.5)<br>Euglena1000: 46.0 (10.7)<br>Euglena3000: 48.0 (9.7)
**Oftedal S, 2019**	Poor sleep quality adult	Age: 18-65 years<br>Shift Workers<br>Inactivity as assessed by the Active Australia Question (MVPA less than 150 minutes per week)<br>Poor dietary quality (6 out of 10 on a brief screening questionnaire)<br>Poor or very poor sleep quality using PSQI<br>Access to an internet-enabled smartphone	20/20	60/45	34.9 (9.2) /36.6 (10.1)
**Wilson D, 2022**	Obesity	Age: 18 years of age or older Pilot with a valid commercial flight license.<br>Pilots working for an airline, full-time BMI of at least 25.	67/58	6.96/10.3	43.7 (10.0) /45.6 (11.4)

Intervention Methods	Intervention	Control	Frequency			Total exercise time	Intensity
			Minutes	Time/week	Total week		
Nutritional intervention	12.5 oz-eq/day group 7.5 oz-eq more than the Dietary Guidelines for Americans protein food group of 5.0 oz-eq/day.<br>An additional 7.5 oz-eq was obtained by increasing the number of servings of animal protein foods (meat, eggs, poultry, and seafood) in the protein food group.	The 5 oz-equivalent (eq) /day group	Non ※1	Non ※1	12 weeks	Non ※1	Non ※1
Nutritional intervention	Euglena ingestion<br>The participants ingested the Midori Mushi powder twice a day (after breakfast and dinner) for 12 weeks.	Placebo (starch) powder was consumed twice daily (after breakfast and dinner) for 12 weeks.	Non ※1	Non ※1	12 weeks	Non ※1	Non ※1
Nutritional intervention and Pysical Activity	Participants used the app to set and self-monitor goals for physical activity (e.g., MVPA minutes, resistance training sessions), diet quality (e.g., number of key foods served, soft drinks/fast food/no alcohol days), and sleep (e.g., time, quality, hygiene, variability (bedtime and wake time)) The participants were asked to submit a weekly summary report.	Habitual Lifestyle	Non ※1	Non ※1	4 weeks	Non ※1	Moderate

Nutritional intervention and Physical Activity	The intervention incorporated Behavior Change Technology.Goals were set between participants and health coaches for (a) sleep hygiene, (b) healthy eating, and (c) Physical Activity.<br>Healthy eating goals were defined based on healthy eating resources.<br>Sleep goals were established based on the Sleep Hygiene Checklist.Physical activity prescription goals were set based on an individual's assessment of physical activity. (150 minutes of moderate intensity and 75 minutes of vigorous intensity per week)	Habitual Lifestyle	Non<br>※1	Non<br>※1	16 weeks	Non<br>※1	Moderateorvigorous

Sleep Measurement Tool, Reference Period, and Outcome Measure	Sleep outcome score				RoB
	Base line (SD)	After intervention (SD)	Amount of change (SD)	Follow-Up (SD)	
The Actigraph (Actiwatch), worn on the wrist, was used to measure at 6 and 12 weeks.<br>Subjective variables of sleep were assessed using a validated questionnaire.<br>PSQI for sleep quality and ESS for subjective sleep duration.	Intervention group<br>Actigraphy<br>Time sleeping,min 374 (13)<br>Sleep efficiency,% 83 (2)<br>WASO, min 39 (4)<br>PSQI 7.6 (0.5)<br>ESS 10.4 (0.8)<br><br>Control group<br>Actigraphy<br>Time sleeping,min 384 (10)<br>Sleep efficiency, % 84 (1)<br>WASO, min 37 (3)<br>PSQI 7.5 (0.4)<br>ESS 9.6 (0.7)	Intervention group<br>Actigraphy<br>Time sleeping,min 378 (12)<br>Sleep efficiency, % 84 (2)<br>WASO, min 39 (4)<br>PSQI 5.1 (0.5)<br>ESS 8.9 (0.8)<br><br>Control group<br>Actigraphy<br>Time sleeping,min 389 (11)<br>Sleep efficiency, % 86 (1)<br>WASO, min 33 (3)<br>PSQI 4.5 (0.4)<br>ESS 7.5 (0.7)	Intervention group<br>Actigraphy<br>Time sleeping,min 4 (9)<br>Sleep efficiency, % 1 (1)<br>WASO, min 0 (2)<br>PSQI -2.5 (0.6)<br>ESS -1.9 (0.7)<br><br>Control group<br>Actigraphy<br>Time sleeping,min 5 (7)<br>Sleep efficiency, % 2 (1)<br>WASO, min -4 (2)<br>PSQI -3.0 (0.5)<br>ESS -2.1 (0.6)	Non	Some concerns
PSQI for sleep quality	Placebo: 7.7 (2.6)<br>Euglena 500: 8.1 (3.1)<br>Euglena 1000: 7.4 (2.4)<br>Euglena 3000: 6.8 (2.2)	Placebo: 6.3 (2.3)<br>Euglena 500: 5.7 (2.3)<br>Euglena 1000: 4.7 (2.1)<br>Euglena 3000: 4.2 (2.1)	Non	4W<br>Placebo: 7.1 (2.6)<br>Euglena 500: 5.9 (2.2)<br>Euglena 1000: 5.8 (2.3)<br>Euglena 3000: 5.1 (2.0)<br><br>8W<br>Placebo: 6.2 (2.4)<br>Euglena 500: 5.5 (1.5)<br>Euglena 1000: 5.4 (2.6)<br>Euglena 3000: 4.9 (2.4)	Some concerns

PSQI for sleep quality	Intervention group<br>7.5 (2 to 18)<br>Control group<br>7.9 (3 to 15)	Intervention group<br>7 (2 to 13)<br>Control group<br>7.1 (3 to 13)	Intervention group<br>-1.0 (-2.5 to 0.4)<br>Control group<br>-0.8 (-2.1 to 0.5)	Non	Some concerns
PSQI for sleep quality	6.4 (2.2) /6.1 (1.9)	4.0 (1.3) /5.8 (1.8)	2.4 (2.0-2.8) /0.3 (0.1-0.5)	Non	High

BCT, Behavior change techniques; BMI, Body Mass Index; I/C, Intervention/Control; ESS, The Epworth Sleepiness Scale; HP, high protein; RP, recommended protein; MVPA , Moderate and Vigorous Physical Activity; PSQI, Participants used the Pittsburgh Sleep Quality Index; RoB, Risk of Bias; SD, Standard deviation; SF-36,MOS 36-Item Short-Form Health Survey; WASO, Wake after sleep onset

※1, Because the intervention method is not exercise
