# Supplementary material for: The effects of nonpharmacological sleep hygiene on sleep quality in nonelderly individuals: A systematic review and network meta-analysis of randomized controlled trials
Source: PLoS One. 2024 Jun 5;19(6):e0301616. doi: 10.1371/journal.pone.0301616 (PMC11152306; doi:10.1371/journal.pone.0301616)
Supplement: S3 Table — (PDF) [file pone.0301616.s004.pdf]

Supplementary Table 3 Detailed summary of lifestyle modification

Author, year	Population	Participant inclusion criteria	N I/C	Female (%) I/C	Mean age<br>I/C
**Ha Y, 2022**	Healthy adult	Age: 19-60 years<br>Nurses with at least 4-night shifts per month in surgical and internal medicine wards	30/27	100/100	27.63 (3.05) /27.03 (3.69)
**Murawski B, 2019**	Poor sleep quality adult	Age: 18-55 year<br>Living in Australia<br>Inadequate physical activity (<90 min/week)<br>Sleep quality "quite poor" or "very poor"	80/80	82.5/77.5	41.1 (9.8) /41.9 (10.1)
**Murawski B, 2020**	Poor sleep quality adult	Age: 18-55 years<br>Physical inactivity (<90 minutes/week)<br>Poor sleep quality (fairly poor or very poor)	80/80	82.5/77.5	41.1 (9.8) /41.9 (10.1)
**Martin CK, 2016**	Healthy adult	Healthy men aged: 20-50 years<br>Healthy women aged: 20-47 years<br>BMI: 22.0-28.0	143/75	69.2/70.6	38.0 (7.3) /37.9 (7.0)
**Fenton S, 2021**	Obesity	Age : 18-65 years<br>The applicant had an iOS/Android smartphone/tablet with internet access and was able to attend all four evaluations.	80/<br>36	71/<br>69	46.3 (10.2) /40.5 (10.7)

Intervention Methods	Detailed intervention	Control	Frequency			Total exercise time	Intensity
			Minutes	Time/week	Total week		
Lifestyle Modification	A mobile wellness program<br>Start Weeks 1-6<br>Goal of 5000 to 9999 steps per day.<br>Week 7-12<br>Add 1000 steps per day every 2 weeks.<br>exercise intensity of 50-60% of target heart rate.<br>Online exercises<br>Twice a week for 1 hour each.	Received Fitbit to self-monitor activity and sleep patterns only.	Start Weeks 1-6<br>Goal of 5000 to 9999 steps per day.<br>Week 7-12<br>Add 1000 steps per day every 2 weeks.	7 time/week	12 weeks	84 times	Vigorous
Lifestyle Modification	Intervention by app (educational resources, self-monitoring, goal setting, feedback) and non-app (intervention delivered via participant handbook, text message, email).	Habitual Lifestyle	Non<br>※1	Non<br>※1	13 weeks	Non<br>※1	Moderate
Lifestyle Modification	encouraged to gradually work towards the amount of weekly physical activity recommended for adults (at least 150min of moderate- or 75 min of vigorous-intensity physical activity, or an equivalent combination, and resistance training on 2 days/week)	Habitual Lifestyle	150 minute	2 time/week	12 weeks	Non<br>※1	Moderate
Lifestyle Modification	2 years of 25% CR	AL control group	Non<br>※1	Non<br>※1	24 Month	Non<br>※1	Non<br>※1
Lifestyle Modification	The enhanced intervention targeted dietary behaviours, physical activity and sleep health.	The traditional intervention targeted dietary and physical activity behaviours	150 min of moderate or 75 min of vigorous intensive physical activity	1 time/ week	24 weeks (6 months)	24 times	moderate or vigorous

Sleep Measurement Tool, Reference Period, and Outcome Measure	Sleep outcome score				RoB
	Base line (SD)	After intervention (SD)	Amount of change (SD)	Follow-Up (SD)	
PSQI for sleep quality	Intervention group<br>PSQI 9.23 (3.18)<br>Control group<br>PSQI 8.73 (3.02)	Intervention group<br>PSQI 7.50 (2.95)<br>Control group<br>PSQI 8.53 (2.82)	Intervention group<br>PSQI -1.70 (3.21)<br>Control group<br>PSQI -0.22 (4.04)	Non	Some concerns
PSQI for sleep quality	Intervention group<br>9.2 (3.07)<br>Control group<br>9.2 (2.86)	Intervention group<br>6.7 (3.04)<br>Control group<br>8.0 (2.34)	Non	24w<br>Intervention group<br>6.3 (2.98)<br>Control group<br>7.5 (2.59)	Some concerns
PSQI for sleep quality	Intervention group<br>7.5 (3.85)<br>Control group<br>8.4 (3.20)	Intervention group<br>6.7 (3.26)<br>Control group<br>8.0 (2.85)	Non	Non	High
PSQI for sleep quality	3.85 (0.22) /3.39 (0.26)	4.07 (2.19) /4.25 (2.92)	0.24 (0.20) /0.60 (0.26)	12 Month<br>3.89 (2.56) /4.26 (3.03)	Some concerns
PSQI for sleep quality.<br>Sleep duration and sleep efficiency were also device-measured using Geneactiv accelerometer data.	Intervention group<br>PSQI 7.1 (3.0)<br>Sleep efficiency 0.86 (0.05)<br>Sleep duration (h/night) 6.4 (0.9)<br>Control group<br>PSQI 6.7 (3.0)<br>Sleep efficiency 0.89 (0.04)<br>Sleep duration (h/night) 6.7 (0.7)	Intervention group<br>PSQI 5.9 (3.6)<br>Sleep efficiency 0.86 (0.05)<br>Sleep duration (h/night) 6.1 (1.0)<br>Control group<br>PSQI 6.4 (3.8)<br>Sleep efficiency 0.88 (0.04)<br>Sleep duration (h/night) 6.4 (0.8)	Non	Non	Some concerns

AL, Ad libitum; BMI, Body Mass Index; CR, Calorie restriction; I/C, Intervention/Control; PSQI, Participants used the Pittsburgh Sleep Quality Index; RoB, Risk of Bias; SD, Standard deviation

※1, Because the intervention method is not exercise
