# Supplementary material for: The effects of nonpharmacological sleep hygiene on sleep quality in nonelderly individuals: A systematic review and network meta-analysis of randomized controlled trials
Source: PLoS One. 2024 Jun 5;19(6):e0301616. doi: 10.1371/journal.pone.0301616 (PMC11152306; doi:10.1371/journal.pone.0301616)
Supplement: S4 Table — (PDF) [file pone.0301616.s005.pdf]

Supplementary Table 4 Detailed summary of exercise

Author, year	Population	Participant inclusion criteria	N I/C	Female (%) I/C	Mean age I/C
**Leonel LDS, 2022**	Obesity	Age: 20-50 years<br>BMI: 30-39.9 kg/m2<br>Not exercising more than twice a week in the past 3 months	NG: 9<br>PG: 13<br>CG: 13	NG: 66.7<br>PG: 53.8<br>CG: 61.5	NG: 37.44 (3.97)<br>PG: 38.00 (6.12)<br>CG: 34.69 (7.45)
**Quist JS, 2019**	Obesity	Age: 20-45 years<br>Caucasian women and men<br>Overweight or Class 1 obese (BMI: 25-35 kg/m2)	BIKE: 22<br>MOD: 30<br>VIG: 24<br>CG: 16	BIKE: 54.5<br>MOD: 50<br>VIG: 54.2<br>CG: 43.8	BIKE: 35 (7)<br>MOD: 32 (7)<br>VIG: 37 (7)<br>CG: 35 (7)
**Tseng TH, 2020**	Poor sleep quality adult	Age: 40 years and older<br>Poor sleep quality (PSQI>5)	20/20	75/90	61.1 (6.8)/62.2 (7.4)
**Niu SF, 2021**	Poor sleep quality adult	Age: Not set<br>Full-time female nursing staff working 8-hour shifts (day, evening, and night shifts) prior to this study<br>Day shift during the study period<br>Worked at least 6 months<br>PSQI score of 5 or higher<br>No regular exercise routine (at least 30 minutes per session, at least 3 times per week) prior to enrollment.	Aerobic exercise: 30<br>Usual Activity: 30	Aerobic exercise: 100<br>Usual Activity: 100	Aerobic exercise: 26.07 (4.03)<br>Usual Activity: 26.03 (4.36)
**Elavsky S, 2007**	Insufficient exercise adults	Age: 42-58 years<br>Sedentary or low activity i.e., exercise less than 30 minutes twice a week at moderate intensity<br>Experienced vasomotor symptoms within 1 month of participation.	Walking: 63<br>Yoga: 61<br>CG: 39	Walking: 100<br>Yoga: 100<br>CG: 100	Walking: 50.5<br>Yoga: 50.0<br>CG: 48.6
**Barrett B, 2020**	Healthy adult	Age: 30-69 years<br>Self-reported average of at least one cold per year or at least two colds in the past year<br>Meet American Heart Association guidelines for fitness for an exercise program	Exercise: 137<br>Meditation: 138<br>Control: 138	Exercise: 78.1<br>Meditation: 76.1<br>Control: 73.2	Exercise: 49.1 (11.4)<br>Meditation: 49.2 (11.2)<br>Control: 50.7 (12.1)

		Willing to participate in either meditation or exercise training (or both by random assignment)<br>Willingness to be immunized against the influenza virus and to undergo routine blood draws, nasal lavage, and medical interview<br>Score 14 or less on the 9-item depression module of PHQ-9<br>Fluency and ability to read and write English well enough to complete the medical questionnaire			
**Atlantis E, 2006**	Healthy adult	Age: Not set<br>Have a physician's permission to begin exercise therapy<br>Able to attend the fitness center for 60 minutes at least 3 days per week and collect physiological data on each of the 3 occasions<br>Willing to be randomly assigned to either the treatment group or the waitlist group	20/24	55.0/54.1	29.0 (7.0) /32.0 (7.0)
**Papp ME, 2019**	Healthy adult	Age: 20-40 years<br>a healthy student residing in Sweden<br>performing physical exercise no more than 2h per week at a moderate intensity or 1h a week at a high intensity.	21/23	14.3/13.0	25 (20-37) /25 (20-39)
**Wang F, 2020**	Healthy adult	Age: 18 years and older.<br>Physically sedentary according to screening..	15/17	73.3/64.7	24.87 (4.91) /<br>23.88 (5.07)
**McDonough DJ, 2022**	Healthy adult	Age: 18-35 years old.<br>Enrolled or working at a university<br>BMI >18.5 kg/m2<br>Physical Activity value in the past month is below the national recommendation (confirmed by questionnaire)	32/32	71.8/78.1	22.69 (3.06) /<br>22.91 (3.68)
**Li M, 2015**	Healthy adult	Age: 18-25 years<br>provided the informed consent; and were freshmen or sophomores.	101/105	85.1/80.0	20.63 (1.03) /<br>20.92 (1.15)
**Ladyodeyse C, 2022**	Healthy adult	Age: 14-19 years<br>Presenting with complaints of non-restorative sleep (poor sleep quality or sleep	18/12	0/0	16.4 (1.6) /16.0 (1.4)

		disturbances) and daytime sleepiness<br>Not participating in an exercise program<br>No physical problems that interfere with exercise performance<br>Not using sleep medications.			
**Ahmadinezhad M, 2017**	postmenopausal	Age : 40-60 years<br>Cessation of menstruation at least 12 months prior to the reference date natural menopause<br>PSQI > 5	PG : 36/<br>AG : 36/<br>CG : 36	PG:100/<br>AG:100/<br>CG:100	PG:51.08 (2.61) /<br>AG:50.25 (3.05) /<br>CG:50.72 (2.51)
**Akinci B, 2022**	insufficient exercise adults	Age：18 - 65 years<br>According to the International Physical Activity Questnaire- Short Form, subjects in the inactivity category (<600 MET-min/week)<br>Able to speak, understand and write in their own language.	Asynchronous group：17/<br>Video-conference group：17	Asynchronous group：76.5/<br>Video-conference group：73.3	Asynchronous group：36.47 (14.39) /<br>Video-conference group：35 (15)

Intervention Methods	Intervention	Control	Frequency			Total exercise time	Intensity
			Minutes	Time/week	Total week		
Aerobic and resistance training	Combined aerobic and resistance training<br>NG: remained at a moderate intensity throughout all study.<br>PG: participated in a linear periodization training, divided into three mesocycles of five weeks each	Did not receive any intervention and was instructed to maintain its routine activities.	Average duration of 60 minutes	3 time/week	16 weeks	48 times	Vigorous
Aerobic exercise	BIKE<br>the intensity was self-chosen and participants were instructed to commute by bike back and forth from work or school<br><br>In MOD and VIG, exercise intensity was prescribed at 50% and 70% VO2peak-reserve, respectively, and target exercise heart rate was adjusted during the intervention at 1.5 and 3 months.	Habitual Lifestyle	Exercise energy expenditure at 320 kcal/day for women and 420 kcal/day for men	5 time/week	30 weeks	150 times	Moderate or vigorous
Aerobic exercise	Each exercise training program consisted of 40 minutes of supervised aerobic exercise and 10 minutes of stretching classes, three times a week for 12 weeks.	Habitual Lifestyle	50-minute exercise sessions	3 time/week	12 weeks	36 times	Vigorous
Aerobic exercise	Continuous walking on a treadmill.<br>Sessions lasted 60 minutes and were conducted three times per week for eight consecutive weeks (24 sessions total). Exercise was performed at a moderate intensity of 60% to 80% of maximal heart rate (formula: [220-age] x 0.6 to 0.8).	The patients maintained their daily routines and did not exercise regularly.	60 minutes	3 times/week	8 weeks	24 times	Vigorous

Walking<br><br>Yoga	Walking Program<br>One hour of moderate-intensity exercise three times a week.<br>Exercise duration began with 15 minutes of sustained exercise and gradually increased to 40 to 45 minutes at the midpoint of the 4-month exercise program.<br>Exercise intensity began at 50% of HRR and increased to 60% to 75% of HRR by the end of the program.<br><br>The yoga program<br>Low intensity, 90-minute meetings were held twice a week.	Participants in the control group received no treatment	Walking Program<br>40-45minutes<br><br><br>The yoga program<br>90-minute	Walking Program<br>3 days/week<br><br><br>The yoga program<br>2 days/week	Walking Program<br>16 weeks<br><br>The yoga program<br>16 weeks	Walking Program<br>48 times<br><br><br>The yoga program<br>32 times	Walking Program<br>vigorous<br><br>The yoga program<br>moderate
Meditation<br><br>Walking	Mindfulness Meditation Training<br>Classes were held weekly for eight weeks.<br>Each class lasted approximately 2.5 hours.<br>Participants were expected to practice for 20 to 45 minutes each day.<br>A five-hour weekend retreat was held during the sixth week.<br><br>Exercise training<br>Similar to mindfulness meditation training<br>Exercise training focused on brisk walking and jogging on treadmills, with customized instruction for those with physical limitations or who did not have access to specific equipment such as stationary	Habitual Lifestyle	180 minutes	1 times/week	8 weeks	8 times	Moderate

	bicycles, road bikes, ellipticals, stairways, or rowing machines.<br>Borg's Perceived Exertion Rating 26 levels of 12 to 16 points.						
Aerobic exercise, resistance training, and education	They performed various aerobic exercises of moderate to high intensity for 20 minutes at least 3 days per week.<br>Subjects also performed full-body weight training exercises of moderate to high intensity for 30 minutes at least 3 days per week.<br>As a behavior change strategy, the health education seminar<br>Topics included "Australian Diseases I," "Australian Diseases II," "Nutrition," "Ergonomics," and "Occupational Jet Lag."	Habitual Lifestyle	20-30 minutes	3 times/week	24 weeks	72 times	Moderate<br>or<br>vigorous
Yoga	Participants performed a standardized 1-h HIY program once a week for six weeks with an instructor; additional home practice was encouraged during the 6-week intervention (weekly reports were collected).	Habitual Lifestyle	60 minutes	1 times/week	6 weeks	6 times	Vigorous
Walking	Participants in the IG were asked to perform DAWE<br>The amount of intervention was targeted at 10,000 steps per day.	CG maintained a sedentary lifestyle	No set times	No set times	4 weeks	No set times	Moderate
Aerobic exercise and resistance training	Aerobic exercise and muscle strengthening PA (physical activity) videos were received weekly.	Weekly general health education videos	6.3(3.9) minutes	1 times/week	12 weeks	12 times	Moderate
Baduanjin	On the basis of their original physical activity habit, participants in the Baduanjin exercise group	Keep their original physical activity habit	60 minutes	5 times/week	12 weeks	60 times	Moderate

	gathered and practiced 1 hour Baduanjin exercise at 5 p.m. each day with a frequency of 5 day per week at the gymnasiums of the university.	during the 12-week intervention period.					
Resistance training	Exercises are performed in 3 sets of 10-12 repetitions<br>The eight exercises were leg extensions, hamstring curls, calf raises, 45° leg press, bench press, biceps curls, triceps pulleys, and lat pulldowns.	No exercise of any kind was performed during the intervention period.	55 minutes	3 times/week	12 weeks	36 times	Vigorous
Pilates<br>Acupressure	PG: 3 sessions of 1 hour per week for 6 consecutive weeks.<br>AG: Received acupressure intervention 3 sessions per week for 6 weeks.	No intervention	PG : 1 hour<br>AG : 5 minutes	3 time/ week	6 weeks.	18 times	moderate
Exercise	The qigong training program was similar for both groups. Relaxed breathing, Relaxing the body, Gathering qi, Self-healing.<br><br>Video-conference group:<br>Exercise sessions lasted 40-45 minutes, 3 days per week for 6 weeks, based on real-time movement demonstrations by the therapist, verbal explanations, and feedback to the subjects.	Asynchronous group: Videos were uploaded to the channel weekly. Subjects were instructed to perform the 6-week qigong training on this channel in the correct sequence, 3 times a week for 6 weeks.	40-45 minutes	3 times / week	6 weeks	18 times	moderate

Sleep Measurement Tool, Reference Period, and Outcome Measure	Sleep outcome score				RoB
	Base line (SD)	After intervention (SD)	Amount of change (SD)	Follow-Up (SD)	
PSQI for sleep quality	NG: 10.55 (0.89)<br>PG: 10.53 (0.94)<br>CG: 10.30 (0.58)	NG: 9.00 (±0.81)<br>PG: 10.15 (±0.64)<br>CG: 9.53 (±0.65)	NG: -1.55<br>PG: -0.38<br>CG: -0.77	Non	Some concerns
Sleep quality (PSQI total score)<br>Sleepiness (ESS total score)	BIKE<br>PSQI: 5 (4; 7) ESS: 7 (4)<br>MOD<br>PSQI: 5 (4; 7) ESS: 8 (3)<br>VIG<br>PSQI: 6 (3; 7) ESS: 7 (4)<br>CG:<br>PSQI: 4 (3; 8) ESS: 7 (3)	BIKE<br>PSQI: 6 (4; 8) ESS: 6 (4)<br>MOD<br>PSQI: 6 (3; 8) ESS: 8 (4)<br>VIG<br>PSQI: 5 (3; 7) ESS: 7 (4)<br>CG:<br>PSQI: 6 (3; 8) ESS: 8 (3)	Non	Non	High
The Actigraph (Actiwatch), worn on the wrist, was used to measure<br><br>PSQI for sleep quality	Exercise Group<br>PSQI: 13.3 (3.9)<br>Actigraph:<br>Sleep efficiency (%): 0.78 (0.12)<br>TST (mins): 338.2 (42.3)<br>WASO (mins): 72.2 (31.8)<br>CG<br>PSQI: 11.5 (3.1)<br>Actigraph:<br>Sleep efficiency (%): 0.74 (0.7)<br>TST(mins): 335.1 (40.3)<br>WASO(mins): 98.6 (31.2)	Exercise Group<br>PSQI: 5.8 (2.9)<br>Actigraph:<br>Sleep efficiency (%): 0.82 (0.06)<br>TST (mins): 357.9 (86.6)<br>WASO (mins): 72.6 (27.7)<br>CG<br>PSQI: 11.0 (3.2)<br>Actigraph:<br>Sleep efficiency (%): 0.73 (0.5)<br>TST(mins): 328.9 (39.4)<br>WASO(mins): 94.5 (29.0)	Non	Non	Some concerns

The Actigraph (Actiwatch), worn on the wrist, was used to measure	Exercise Group<br>Actigraph:<br>TST (mins): 364.80 (79.14)<br>Sleep onset latency (mins): 10.01 (13.25)<br>WASO (mins): 24.31 (27.43)<br>Sleep efficiency (%): 89.88 (6.60)<br>CG<br>Actigraph:<br>TST (mins): 378.46 (68.85)<br>Sleep onset latency (mins): 11.31 (21.55)<br>WASO (mins): 22.82 (28.08)<br>Sleep efficiency (%): 90.27 (8.02)	Exercise Group<br>Actigraph:<br>TST (mins): 421.42 (91.51)<br>Sleep onset latency (mins): 5.48 (5.62)<br>WASO (mins): 21.75 (21.97)<br>Sleep efficiency (%): 93.11 (4.56)<br>CG<br>Actigraph:<br>TST (mins): 368.13 (91.71)<br>Sleep onset latency (mins): 12.34 (11.20)<br>WASO (mins): 23.45 (23.81)<br>Sleep efficiency (%): 89.52 (6.32)	Non	16w<br>Exercise Group<br>Actigraph:<br>TST (mins): 405.25 (73.48)<br>Sleep onset latency (mins): 8.19(9.94)<br>WASO (mins): 21.96 (31.96)<br>Sleep efficiency (%): 91.33 (6.41)<br>CG<br>Actigraph:<br>TST (mins): 373.78 (64.61)<br>Sleep onset latency (mins): 9.94 (14.77)<br>WASO (mins): 25.89 (35.58)<br>Sleep efficiency (%): 89.04(9.08)	Some concerns
PSQI for sleep quality	Walking: 6.02 (3.17)<br>Yoga: 6.90 (3.94)<br>CG: 5.46 (2.96)	Walking: 4.97 (2.74)<br>Yoga: 6.48 (4.22)<br>CG: 5.44 (3.63)	Non	Non	Some concerns
PSQI for sleep quality	Exercise: 6.2 (3.6)<br>Meditation: 5.8 (3.3)<br>CG: 5.7 (3.3)	Exercise: 5.11 (2.36)<br>Meditation: 5.57 (2.36)<br>CG: 6.10 (2.37)	Exercise: 0.98 (-0.41, -1.56)<br>Meditation: 0.53 (-0.04, 1.10)<br>CG: Non	3 months<br>Exercise: 5.15 (2.34)<br>Meditation: 5.16 (2.32)<br>CG: 5.89 (2.35)<br>5 months<br>Exercise: 5.18 (2.36)<br>Meditation: 5.28 (2.35)<br>CG: 5.76 (2.36)	Some concerns

				7 months<br>Exercise: 5.12 (2.37)<br>Meditation: 5.18 (2.36)<br>CG: 5.96 (2.36)	
PSQI for sleep quality	6.0 (2.5) /6.6 (3.2)	4.1 (1.8) /5.4 (2.6)	-2.0 (2.6)/-1.3 (2.7)	Non	Some concerns
PSQI for sleep quality<br>ISI<br>Measure the subjective symptoms and consequences of insomnia and the degree of anxiety and distress caused by sleep disturbances.	PSQI<br>5.5 (3.2) /5.4 (2.3)<br>ISI<br>8.3 (6.1) /7.3 (3.6)	PSQI<br>5.7 (3.7) /4.8 (1.8)<br>ISI<br>8.0 (6.2)/6.5 (3.7)	Non	Non	High
PSQI for sleep quality	5.6 (3.46) /4.58 (2.02)	5.14 (2.60) /5.42 (2.39)	Non	Non	High
The Actigraph (Actiwatch), worn on the wrist, was used to measure	Sleep duration (h/night)<br>7.99 (0.82) /8.07 (0.74)<br>Sleep efficiency (%)<br>84.62 (7.12) /86.69 (5.99)	Sleep duration (h/night)<br>8.07 (0.75) /7.84 (1.41)<br>Sleep efficiency (%)<br>88.44 (4.83) /85.88 (6.68)	Non	Non	Some concerns
PSQI for sleep quality	3.67 (1.48) /4.08 (1.73)	4.77 (1.78) /5.12 (1.66)	1.09 (1.87) /1.05 (1.64)	24 weeks<br>3.56 (1.62) /3.79 (1.80)	Some concerns
PSQI for sleep quality	6.3 (0.8)/7.3 (0.7)	7.4 (0.7)/5.1 (0.6)	Non	Non	Some concerns
PSQI for sleep quality	PG<br>PSQI 12.05 (2.82)<br>AG<br>PSQI 14.80 (2.08)<br>CG<br>PSQI 14.69 (2.27)	PG<br>PSQI 4.75 (2.62)<br>AG<br>PSQI 7.48 (3.25)<br>CG<br>PSQI 14.58 (2.90)	PG<br>PSQI −7.30 (2.40)<br>AG<br>PSQI −7.31 (3.53)<br>CG<br>PSQI −0.11 (2.22)	Non	Some concerns
PSQI for sleep quality	Asynchronous group：5.94 (2.51) / Video-conference group：6.71 (2.14)	Asynchronous group：4.71 (1.92) / Video-conference group：4.59 (2.12)	Asynchronous group：-2.50 (0.03) / Video-conference group：-3.32 (0.91)	Non	Some concerns

AG, Acupressure Group; BIKE, Active commuting by bike; BMI, Body Mass Index; CG, Control; DAWE, Daily aerobic walking exercise; ESS, The Epworth Sleepness Scale; HIY, high intensity hatha yoga exercises; HRR, Heart rate reserve; I/C, Intervention/Control; IG, Intervention group; ISI, The Insomnia Severity Index; MOD, Leisuretime exercise of moderate intensity; NG, Non-periodized; PG, Pilates Group; PG, Periodized; PHQ-9, The Patient Health Questionnaire; PSQI, Participants used the Pittsburgh Sleep Quality Index; RoB, Risk of Bias; SD, Standard deviation; TST, Total sleep time; VIG, Vigorous intensity; WASO, Wake after sleep onset
