# Supplementary material for: The effects of nonpharmacological sleep hygiene on sleep quality in nonelderly individuals: A systematic review and network meta-analysis of randomized controlled trials
Source: PLoS One. 2024 Jun 5;19(6):e0301616. doi: 10.1371/journal.pone.0301616 (PMC11152306; doi:10.1371/journal.pone.0301616)
Supplement: S5 Table — (PDF) [file pone.0301616.s006.pdf]

Supplementary Table 5　Detailed summary of physical activity

Author, year	Population	Participant inclusion criteria	N I/C	Female (%) I/C	Mean age<br>I/C
**Genin PM, 2017**	Healthy adult	Age: Not set<br>Company employees who were requested to participate through the manufacturer's internal network<br>Those who received the information sheet and signed the consent form as requested by the ethics authorities.	Expert: 36<br>Novice: 37<br>Control: 22	34.74<br>※1	44 (9.9)<br>※1
**Rayward AT, 2020**	Poor sleep quality adult	Age: 40-65 years<br>Respondents reporting fairly poor or very poor sleep quality (PSQI)<br>MVPA less than 90 minutes/week.<br>BMI: 18.5-35.0<br>Had internet access and mobile app access on a device compatible with the intervention.	PAS: 110<br>SO: 110<br>PI: 220<br>Control: 55	PAS: 80.1<br>SO: 83.6<br>PI: 84.5<br>Control: 82.9	PAS: 51.7 (6.7)<br>SO: 52.2 (7.0)<br>PI: 51.9 (6.9)<br>Control: 52.4 (7.3)
**Tadayon M, 2016**	Postmenopausal	Age: Not set<br>Postmenopausal women<br>No menstruation for 1 year<br>who had menopause confirmed by blood tests to check follicle stimulating hormone and luteinizing hormone levels<br>Those with sleep disturbances as assessed by PSQI	56/56	100/100	52.3 (1.6)/52.48 (1.7)
**Hurdiel R, 2017**	Healthy adult	Age: 18-24 years old<br>PSQI score of 5 or higher<br>TST less than 7 hours per day and sleep latency greater than 30 minutes<br>Practiced less than 1 hour per week of moderate or intense Physical Activity level as determined by GPAQ	10/9	100/100	20.1 (1.7)<br>※1

Intervention Methods	Intervention	Control	Frequency			Total exercise time	Intensity
			Minutes	Time/week	Total week		
Physical activity	Two training sessions per week.<br>Each session is a minimum of 45 minutes and alternates between muscle strengthening exercises and aerobic breathing exercises (once a week).<br>Participants could also add a collective activity as a third session each week.	No intervention	Minimum of 45 minutes	2 time/week	20 weeks	40 times	Vigorous
Sleep Hygiene and Physical Activity	Sleep intervention<br>reducing bed and wake time variability, engaging in a number of sleep hygiene behaviors stress management<br><br>Physical Activity<br>increasing their daily minutes of MVPA and step counts and weekly RT	Habitual Lifestyle	Non<br>※2	Non<br>※2	24 weeks	Non<br>※2	Moderate
Physical activity	Walking with a pedometer<br>Participants were asked to start by walking a distance that was comfortable for them, adding 500 steps each week to reach a maximum of 10,000 steps per day by the end of the 12 weeks.	Habitual Lifestyle	Non<br>※2	Non<br>※2	12 weeks	Non<br>※2	Moderate
Physical Activity	For 12 weeks, they practiced moderate-intensity physical activity together for 1.5 hours twice a week at 6 pm, according to WHO recommendations. Physical activity was supervised by the second author (researcher and sports educator) and included roller skating, biking, baseball, and walking/running.	No activity intervention	90 minutes	2 times/week	12 weeks	24 times	Moderate

Sleep Measurement Tool, Reference Period, and Outcome Measure	Sleep outcome score				RoB
	Base line (SD) I/C	After intervention (SD) I/C	Amount of change (SD)	Follow-Up (SD) I/C	
ISI<br>Measure the subjective symptoms and consequences of insomnia and the degree of anxiety and distress caused by sleep disturbances.<br><br>Somnolence Scale: ESS<br>measuring daytime sleepiness in adults	Expert<br>ISI: 6.3 (3.4) ESS: 6.5 (3.4)<br>Novice<br>ISI: 9.1 (4.7) ESS: 8 (3.2)<br>Control<br>ISI: 8.9 (4.9) ESS: 7.5 (3.3)	Expert<br>ISI: 5.2 (3.4) ESS: 6.3 (3.4)<br>Novice<br>ISI: 6.1 (3.8) ESS: 7 (2.7)<br>Control<br>ISI: 7.7 (4.6) ESS: 8.6 (3.6)	Non	Non	High
PSQI for sleep quality	PAS: 9.84 (3.06)<br>SO: 10.39 (3.11)<br>PI: 10.11 (3.09)<br>Control: 10.67 (3.05)	PAS: 7.53 (3.92)<br>SO: 7.23 (3.61)<br>PI: 7.39 (3.77)<br>Control: 9.91 (3.99)	Non	3 months<br>PAS: 7.68 (3.83)<br>SO: 7.85 (3.17)<br>PI: 7.76 (3.53)<br>Control: 10.10 (3.54)	High
PSQI for sleep quality	12.69 (2.41)/13.05 (1.61)	9.35 (3.01)/13.05 (1.62)	Non	4th week<br>11.76 (2.61)/9.89 (3.07)<br>8th week<br>13.23 (1.57)/13.14 (1.54)	Some concerns
The Actigraph (Actiwatch), worn on the wrist, was used to measure<br><br>PSQI for sleep quality	PSQI<br>9.1 (1.7)/10.5 (3.2)<br><br>Actigraph:<br>TST(mins)<br>475 (32)/482 (42)	PSQI<br>4.8 (2.0)/8.7 (2.9)<br><br>Actigraph:<br>TST (mins)<br>486 (33)/460 (38)	Non	6weeks<br>PSQI<br>6.0 (1.8)/8.4 (2.7)	Some concerns

BMI, Body Mass Index; ESS, The Epworth Sleepness Scale; GPAQ, The Global Physical Activity Questionnaire; I/C, Intervention/Control; ISI, The Insomnia Severity Index; MVPA, Moderate and Vigorous Physical Activity; PAS, Physical Activity and Sleep Health group; PI, Pooled Intervention groups; PSQI, Participants used the Pittsburgh Sleep Quality Index; RT, Resistance Training; RoB, Risk of Bias; SD, Standard deviation ; SO, Sleep Health Only group; TST, Total sleep time; WHO, World Health Organization

※1, No indication by group

※2, Because the intervention method is not exercise
