# Supplementary material for: The effects of nonpharmacological sleep hygiene on sleep quality in nonelderly individuals: A systematic review and network meta-analysis of randomized controlled trials
Source: PLoS One. 2024 Jun 5;19(6):e0301616. doi: 10.1371/journal.pone.0301616 (PMC11152306; doi:10.1371/journal.pone.0301616)
Supplement: S6 Table — (PDF) [file pone.0301616.s007.pdf]

Supplementary Table 6 P-score

	P-score
res	0.9998
pa	0.8565
nut	0.8335
nutpa	0.7643
sh	0.5684
plb	0.5484
shpa	0.5182
aerresed	0.4964
med	0.4290
aer	0.4134
lsm	0.3994
ed	0.3311
aerres	0.3225
bad	0.3178
self	0.2773
yoga	0.2528
ctrl	0.1713

aer, aerobic exercise; aerres, aerobic exercise and resistance training; aerresed, aerobic exercise, resistance training, and education; bad, baduanjin; ctrl, control: ed, education; lsm, lifestyle modification; med, meditation; nut, nutritional intervention; nutpa, nutritional intervention and pysical activity; pa, physical activity; plb, placebo; res, resistance training; self, self-monitor; sh, sleep hygiene; shpa, sleep hygiene and physical activity
