# Supplementary material for: The effects of nonpharmacological sleep hygiene on sleep quality in nonelderly individuals: A systematic review and network meta-analysis of randomized controlled trials
Source: PLoS One. 2024 Jun 5;19(6):e0301616. doi: 10.1371/journal.pone.0301616 (PMC11152306; doi:10.1371/journal.pone.0301616)
Supplement: S7 Table — (PDF) [file pone.0301616.s008.pdf]

Supplementary Table 7 Risk of Bias

	Hudson JL, 2020	Nakashima A, 2020	Oftedal S, 2019	Wilson D, 2022	Ha Y, 2022	Murawski B, 2019	Murawski B, 2020	Martin CK, 2016	Fenton S, 2021
1. Bias arising from the randomization process	?	?	+	×	+	+	?	+	?
2. Bias due to deviations from intended interventions.	?	?	?	×	?	?	?	+	+
3. Bias due to missing outcome data.	+	+	+	+	+	+	+	+	+
4. Bias in measurement of the outcome.	?	+	?	+	?	+	×	?	+
5. Bias in selection of the reported result.	?	?	?	+	?	+	×	?	+
Overall	?	?	?	×	?	?	×	?	?

Judgement

×	High
?	Some concerns
+	Low

	Leonel LDS, 2022	Quist JS, 2019	Tseng TH, 2020	Niu SF, 2021	Elavsky S, 2007	Barrett B, 2020	Atlantis E, 2006	Papp ME, 2019	Wang F, 2020
1. Bias arising from the randomization process	+	?	+	+	+	+	?	?	?
2. Bias due to deviations from intended interventions.	?	×	?	+	?	?	+	×	×
3. Bias due to missing outcome data.	+	+	+	+	+	+	+	+	+
4. Bias in measurement of the outcome.	+	?	?	?	+	+	?	+	×
5. Bias in selection of the reported result.	?	?	?	?	?	+	?	×	?
Overall	?	×	?	?	?	?	?	×	×

Judgement

×	High
?	Some concerns
+	Low

	McDonough DJ, 2022	Li M, 2015	Santiago LCS, 2022	Ahmadinezhad M, 2017	Akinci B, 2022	Genin PM, 2017	Rayward AT, 2020	Tadayon M, 2016	Hurdiel R, 2017
1. Bias arising from the randomization process	?	+	?	+	+	×	+	?	?
2. Bias due to deviations from intended interventions.	+	+	×	?	?	?	+	?	?
3. Bias due to missing outcome data.	+	+	+	+	+	+	+	+	+
4. Bias in measurement of the outcome.	+	+	?	+	+	?	?	?	+
5. Bias in selection of the reported result.	?	?	?	?	?	?	×	?	?
Overall	?	?	?	?	?	×	×	?	?

Judgement

Symbol	Judgement
×	High
?	Some concerns
+	Low
