# Supplementary material for: The effects of nonpharmacological sleep hygiene on sleep quality in nonelderly individuals: A systematic review and network meta-analysis of randomized controlled trials
Source: PLoS One. 2024 Jun 5;19(6):e0301616. doi: 10.1371/journal.pone.0301616 (PMC11152306; doi:10.1371/journal.pone.0301616)
Supplement: S8 Table — (PDF) [file pone.0301616.s009.pdf]

Supplementary Table 8 GRADE assessment

Comparison		Direct estimate				Network estimate			
Treatment 1	Treatment 2	**Odds ratio (95% confidence interval)**	**Quality of evidence**	**Final rating**		Odds ratio (95% confidence interval)	**Quality of evidence**	**Final rating**	
aer	ctrl	2.18 (0.98 to 5.11)	⊕○○○	VERY LOW	a,c				
aer	med					0.94 (0.36 to 2.52)	⊕○○○	VERY LOW	a,c
aer	yoga					1.81 (0.28 to 11.57)	⊕○○○	VERY LOW	a,c
aerres	ctrl	1.46 (0.59 to 3.55)	⊕○○○	VERY LOW	a,b,c				
aerresed	ctrl	2.76 (0.25 to 29.71)	⊕○○○	VERY LOW	a,c				
bad	ctrl	1.43 (0.61 to 3.37)	⊕○○○	VERY LOW	a,c				
lsm	ctrl	1.82 (0.76 to 4.35)	⊕○○○	VERY LOW	a,b,c				
med	ctrl	1.49 (0.54 to 4.12)	⊕⊕○○	LOW	c				
nut	ctrl					11.16 (6.96 to 17.88)	⊕○○○	VERY LOW	a,c
nutpa	ctrl	7.91 (2.92 to 21.83)	⊕○○○	VERY LOW	a,b,c				
pa	ctrl	15.75 (3.07 to 80.59)	⊕○○○	VERY LOW	a,c				
res	ctrl	561.25 (239.29 to 1316 38)	⊕○○○	VERY LOW	a,c				
sh	ctrl	3.55 (0.57 to 22.23)	⊕○○○	VERY LOW	a,c				
shpa	ctrl	2.97 (0.29 to 30.26)	⊕○○○	VERY LOW	a,c				
yoga	ctrl					1.04 (0.16 to 6.60)	⊕○○○	VERY LOW	a,c
ed	pa	0.06 (0.00 to 3.90)	⊕○○○	VERY LOW	a,c				
lsm	self	1.89 (0.12 to 28.66)	⊕○○○	VERY LOW	a,c				
nut	plb	3.25 (0.37 to 28.66)	⊕○○○	VERY LOW	a,c				
sh	shpa	1.16 (0.21 to 6.48)	⊕○○○	VERY LOW	a,c				

Odds ratios were estimated using standardized mean differences.

(a) risk of bias (some of the included studies were classified like ‘deficient by the PEDro scale); (b) inconsistency (the included studies used different measurement instrument); (c) imprecision (the sample size was small).
