# Supplementary figures and images for: The effects of nonpharmacological sleep hygiene on sleep quality in nonelderly individuals: A systematic review and network meta-analysis of randomized controlled trials
Source: PLoS One. 2024 Jun 5;19(6):e0301616. doi: 10.1371/journal.pone.0301616 (PMC11152306; doi:10.1371/journal.pone.0301616)

Supplementary Figure 1

a net-heat plot

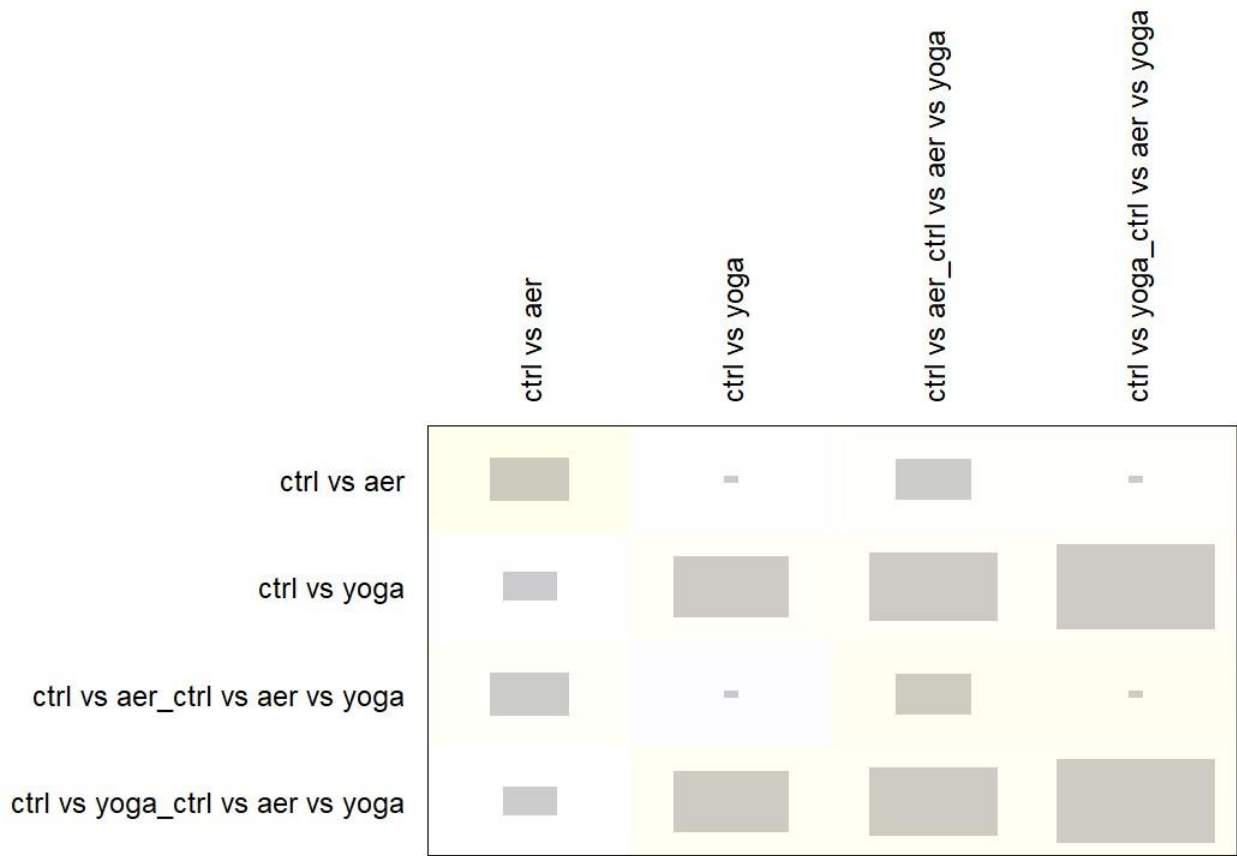

aer, aerobic exercise; ctrl, control

Supplement: S1 Fig — (PDF) [file pone.0301616.s010.pdf]
